# Supplementary material for: Serotonin modulates a depression-like state in Drosophila responsive to lithium treatment
Source: Nat Commun. 2017 Jun 6;8:15738. doi: 10.1038/ncomms15738 (PMC5467214; doi:10.1038/ncomms15738)
Supplement: Supplementary Information — Supplementary Figures, Supplementary Tables and Supplementary References [file ncomms15738-s1.pdf]

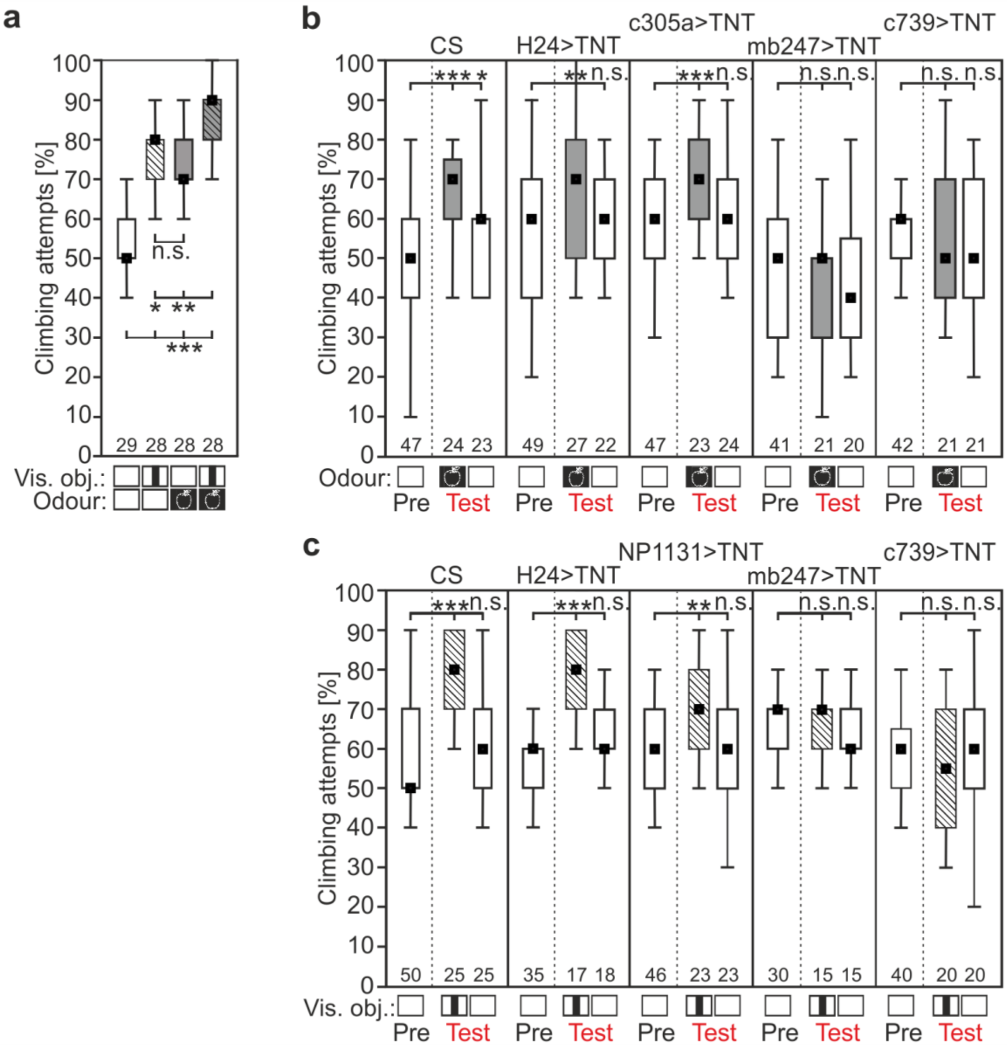

Supplementary Figure 1 | Visual and odour stimuli enhance the motivation for trying to overcome a broad gap via mushroom body α-/β-lobes. Rate of climbing initiation of CS males at a just insurmountable gap of 4.5 mm width. Ten approaches to the gap per male fly. (**a**) An attractive visual landmark (dark vertical stripe with contact to the ground) as well as an attractive odour (yeast-apple juice) coming from the distal side of the gap enhance the fraction of climbing attempts as compared to a featureless climbing block. The combination of odour and visual cues is most effective. (**b**) Mapping of the odour-induced enhancement of climbing initiation by selective blocking the chemical synapses of groups of mushroom body Kenyon cells by UAS-TNT expression[S1]; wild-type CS for comparison. After a pre-test without odour half of the flies within a test group were tested 24h later and starved for 16h again without odour and the other half with yeast-apple juice odour coming from the distal side. A predominant block of γ-lobe neurons (*H24*-GAL4) or α'-/β'-lobes (*c305a*-GAL4)[S2] does not impede the odour-induced increase of motivation. A predominant block of α-/β-/γ-lobes (*mb247*-GAL4) or just α-/β-lobes (*c739*-GAL4) precludes enhancement of motivation. (**c**) Corresponding experiment to (b) for mapping the

visually induced enhancement of climbing initiation by a dark vertical stripe behind the distal gap-side. Again, blocking of γ-lobes (*H24*-GAL4) or α'-/β'-lobes (*NP1131*-GAL4)[S2] leaves the motivational enhancement intact. A predominant block of α-/β-/γ-lobes (*mb247*-GAL4) or just α-/β-lobes (*c739*-GAL4) precludes visual enhancement of motivation. Black squares, medians; boxes, 25% and 75% quartiles; whiskers, 10% and 90% quantiles; numbers of animals tested are indicated below the boxes; n.s., not significant; *, $p<0.05$; **, $p<0.01$; ***, $p<0.001$; Mann-Whitney-U test.

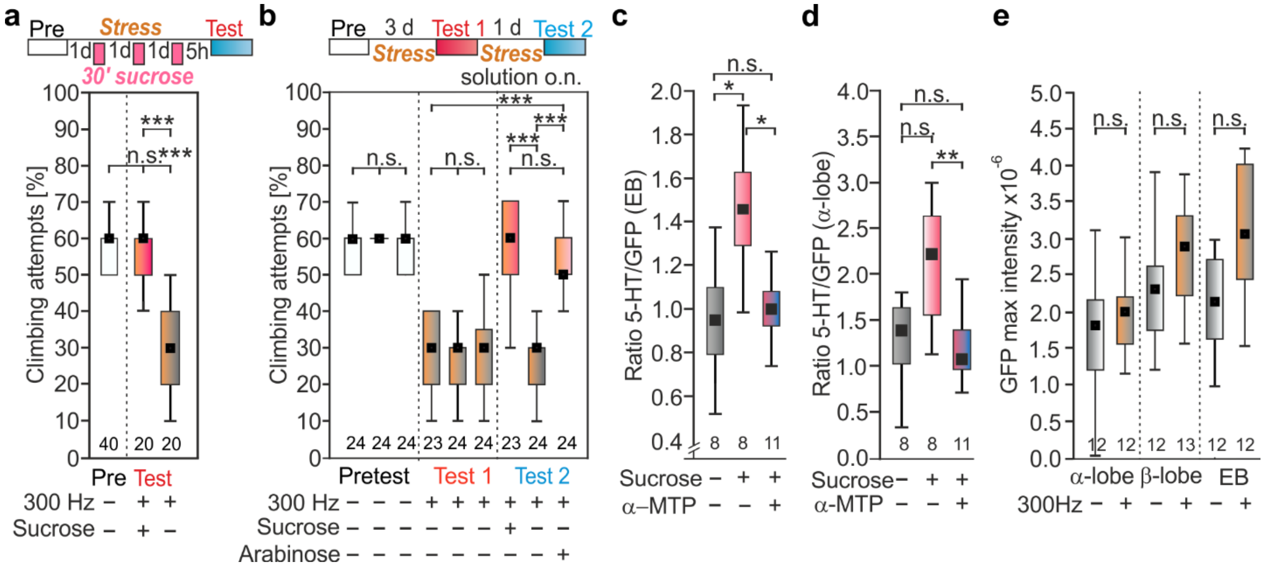

Supplementary Figure 2 | Sweet sensation can prevent or relief the depression like state. (**a**) After each full-day vibration treatment (10 h), flies were put on 5% sucrose solution for 30 min and thereafter on regular fly food overnight. This daily treatment prevents the depression-like state. (**b**) Treating stressed flies overnight with either 5% sucrose or 5% arabinose relieves them from the depressive-like state. Note that arabinose has no caloric value to *Drosophila* suggesting that the sense of sweet can ameliorate the depressive-like state. (**c-d**) Sucrose offered overnight increases the 5-HT level in the entire brain in comparison to flies left overnight on water. 5-HT level does not increase when the blocker of 5-HT synthesis α-methyl-DL-tryptophan (α-MTP; 20mM) is added to the sucrose solution. 5-HT label was quantified from confocal stacks stained with 5-HT antibody of *189Y*-GAL4>UAS-mCD8::GFP flies. In the ellipsoid-body region layers with the broadest GFP expression were sampled in 22.5 µm z-projections. At the α-lobes of the mushroom body all layers expressing GFP were sampled (see Fig. 4c). (**e**) *189Y*-GAL4 driven GFP expression does not change significantly upon stress application (control for 5-HT measurements in Fig.4d; z = 22.5 µm). Black squares, medians; boxes, 25% and 75% quartiles; whiskers, 10% and 90% quantiles; numbers of animals tested are indicated below the boxes. n.s., not significant; *, $p<0.05$; **, $p<0.01$; ***, $p<0.001$; Kruskal-Wallis test.

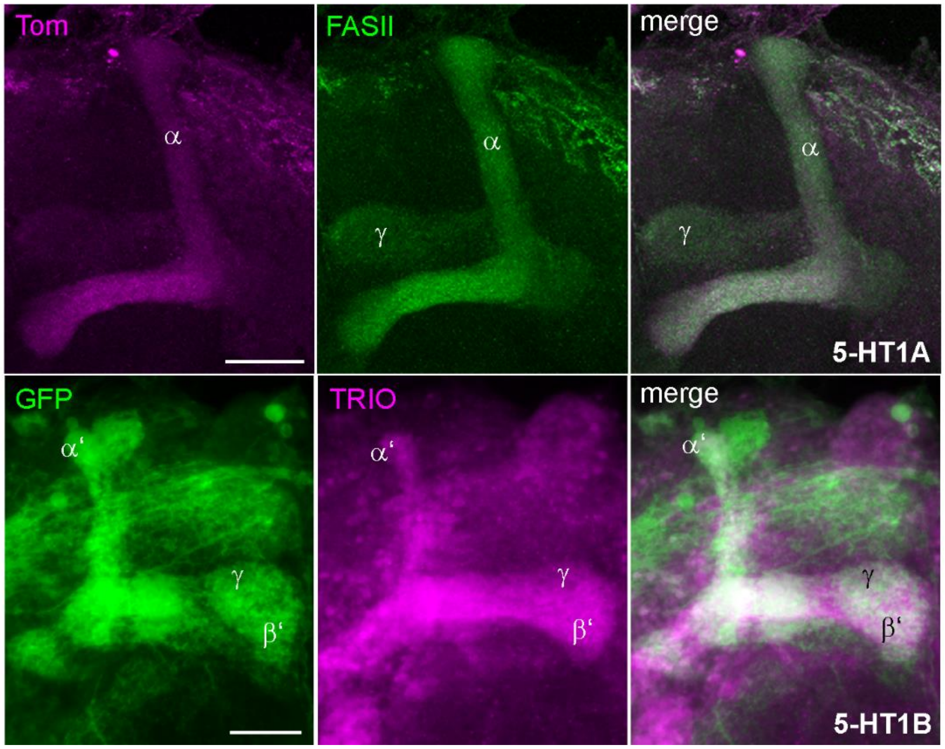

Supplementary Figure 3 | Expression analysis of the 5-HT-1A and 5-HT-1B driver lines. (**a**) The UAS-CD4::tandem-Tomato (Tom; magenta) was used to report the expression pattern of the 5-HT-1A-GAL4 driver line[S3]. A weak but specific signal is detected in the α-/β-Kenyon cells of the mushroom body (52.5 µm z-projection). Anti-Fasciclin II (FASII; green) was used to visualize the α-/β-lobes (and weakly the γ-lobes) of the mushroom body. (**b**) Expression of mCD8-GFP (GFP; green) in the pattern of the 5-HT-1B driver line[S4] is detected in the α'-/β'- and γ-Kenyon cells of the mushroom body (54.75 µm z-projection). Anti-TRIO (magenta) was used to visualize the α'-/β'- and γ-lobes) of the mushroom body. Scale bars denote 25µm.

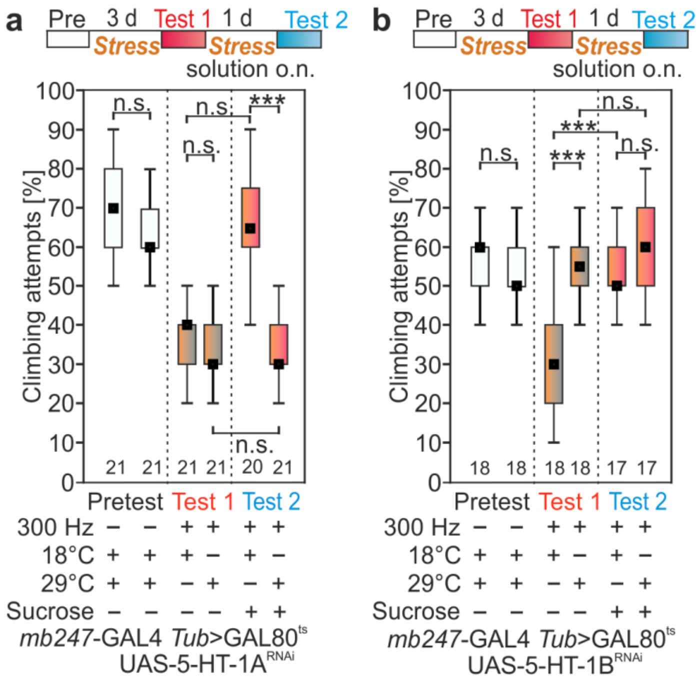

Supplementary Figure 4 | Conditional knock down of 5-HT-1A and 5HT-1B receptor genes in the adult mushroom body. To restrict the RNAi mediated knock downs to the adult stage the *mb247*-GAL4 driver line (expresses in the α-/β- and y-Kenyon cells of the mushroom body[S2]) was combined with the ubiquitously expressed temperature-sensitive GAL4 repressor transgene *Tub*>GAL80^{ts} (ref. S5). Flies were raised at the restrictive temperature of 18°C and tested for the motivation to climb at the 4.5mm gap (Pretest). After each day of stress application (~22°C lab temperature) the experimental group was shifted to 29°C overnight, whereas the controls were set on 18°C again. (**a**) Knock down of 5-HT-1A receptor gene expression (#25834) in the adult mushroom body prevents the sucrose relief of the depressive-like state. (**b**) Knock down of 5-HT-1B receptor gene expression (#27634) in the adult mushroom body conveys resilience to the vibration stress. Therefore feeding sucrose to the stressed flies changes the motivation to climb only in the control (18°C) group. Black squares, medians; boxes, 25% and 75% quartiles; whiskers, 10% and 90% quantiles; numbers of animals tested are indicated below the boxes. n.s., not significant; *, p<0.05; **, p<0.01; ***, p<0.001; Kruskal-Wallis test.

Supplementary Table 1 | Statistical analysis for Figure 1.

Tab. 1a refers to data presented in Figure 1a.

p-values of the KOLMOGOROV-SMIRNOV-Test (KS); LILLIFORS-Tests (Lillif) and SHAPIRO-WILK-Test (SW)

	N	Std.Dev.	p KS	p Lillif	p SW
**Pretest stressed group**	50	0.7825	< 0.01	< 0.01	$< 10^{-5}$
**Pretest control group**	50	0.7071	< 0.01	< 0.01	$< 10^{-5}$
**Test 1 stressed group**	20	1.3018	< 0.20	< 0.01	= 0.0606
**Test 1 control group**	20	1.1743	< 0.15	< 0.01	= 0.1166
**Test 2 stressed group**	15	1.7182	> 0.20	> 0.20	= 0.7916
**Test 2 control group**	14	0.9972	> 0.20	< 0.05	= 0.1737
**Test 3 stressed group**	15	1.5523	< 0.20	< 0.01	= 0.0382
**Test 3 control group**	15	1.3558	> 0.20	< 0.15	= 0.1543

Comparison of the unpaired median data by the MANN-WHITNEY-U-Test (MannU) at the individual days.

Group	U	Z	2*1 sided exact p
**Pretest stress vs. control**	1159.00	-0.6273	0.5343
**Test 1 stress vs. control**	195.50	-0.1217	0.9042
**Test 2 stress vs. control**	79.00	-1.1347	0.2703
**Test 3 stress vs. control**	37.00	-3.1316	0.0012

Comparison of the unpaired median data by the KRUSKAL-WALLIS-TEST (K-W).
Upper row: multiple p-values; lower row: z-values

	(2)	(3)	(4)	(5)	(6)	(7)	(8)
K-Wallis-Test: H(7 N=199)=37.4259 $p < 10^{-5}$							
**Pretest/stressed (1)**<br>R: 111.93	~1.0000<br>0.5444	~1.0000<br>0.6337	~1.0000<br>0.4745	0.2633<br>2.5970	~1.0000<br>1.4234	$< 10^{-5}$<br>4.6516	~1.0000<br>0.6270
**Pretest/control (2)**<br>R: 118.20	--	~1.0000<br>1.0452	~1.0000<br>0.8860	0.0843<br>2.9668	~1.0000<br>1.7835	$< 10^{-5}$<br>5.0214	~1.0000<br>0.9968
**Test 1/stressed (3)**<br>R: 102.28	--	--	~1.0000<br>0.1332	~1.0000<br>1.7475	~1.0000<br>0.7540	0.0122<br>3.5183	~1.0000<br>0.0496
**Test 1/control (4)**<br>R: 104.70	--	--	--	~1.0000<br>1.8708	~1.0000<br>0.8749	0.0076<br>3.6416	~1.0000<br>0.1728
**Test 2/stressed (5)**<br>R: 67.90	--	--	--	--	~1.0000<br>0.8991	~1.0000<br>1.6564	~1.0000<br>1.5883
**Test 2/control (6)**<br>R: 87.143	--	--	--	--	--	0.3223<br>2.5268	~1.0000<br>0.6615
**Test/stressed (7)**<br>R: 33.067	--	--	--	--	--	--	0.0329<br>3.2447
**Test/control (8)**<br>R: 101.30	--	--	--	--	--	--	--

Tab. 1b refers to data presented in Figure 1b.

p-values of the Kolmogorov-Smirnov-Test (KS); Lillifors-Tests (Lillif) and Shapiro-Wilk-Test (SW)

Group	N	Std.Dev.	p KS	p Lillif	p SW
**stressed**	46	13.3221	> 0.20	< 0.15	= 0.0224
**control**	51	12.0736	> 0.20	> 0.20	= 0.7206

Comparison of the unpaired median data by the Mann-Whitney-U-Test (MannU) of the walking activity of the stressed and the control group

Group	U	Z	2*1 sided exact p
**stressed vs. control**	772.00	-2.8971	0.0035

Tab. 1c refers to data presented in Figure 1c.

p-values of the Kolmogorov-Smirnov-Test (KS); Lillifors-Tests (Lillif) and Shapiro-Wilk-Test (SW)

Group	N	Std.Dev.	p KS	p Lillif	p SW
**stressed**	33	233.4105	< 0.05	< 0.01	= 0.0001
**control**	34	189.6690	< 0.05	< 0.01	$< 10^{-5}$

Comparison of the unpaired median data by the Mann-Whitney-U-Test (MannU) of latency time (courtship activity) of the stressed and the control group

Group	U	Z	2*1 sided exact p
**stressed vs. control**	314.50	3.0914	0.0017

Tab. 1d refers to data presented in Figure 1d.

p-values of the Kolmogorov-Smirnov-Test (KS); Lillifors-Tests (Lillif) and Shapiro-Wilk-Test (SW)

	N	Std.Dev.	p KS	p Lillif	p SW
**stressed**	63	24.5805	< 0.01	< 0.01	$< 10^{-5}$
**control**	74	19.1411	< 0.01	< 0.01	$< 10^{-5}$

Comparison of the unpaired median data by the Mann-Whitney-U-Test (MannU) of light transitions (fast phototaxis) of the stressed and the control group

Group	U	Z	2*1 sided exact p
**stressed vs. control**	2231.50	0.4297	0.6684

Tab. 1e refers to data presented in Figure 1e.

p-values of the Kolmogorov-Smirnov-Test (KS); Lillifors-Tests (Lillif) and Shapiro-Wilk-Test (SW)

Group	N	Std.Dev.	p KS	p Lillif	p SW
**stressed**	15	0.0582	> 0.20	> 0.20	= 0.5690
**control**	15	0.0546	> 0.20	> 0.20	= 0.9813

Comparison of the normally distributed data by the unpaired T-Test (K-W) of the optomotor response of the stressed and the control group.

Group	T	F	p
**stressed vs. control**	-0.4531	1.1358	0.6540

Comparison of the unpaired median data by the MANN-WHITNEY-U-Test (MannU) of the optomotor response of the stressed and the control group.

Group	U	Z	2*1 sided exact p
**stressed vs. control**	102.50	0.4148	0.6827

Tab. 1f refers to data presented in Figure 1f.

p-values of the KOLMOGOROV-SMIRNOV-Test (KS); LILLIFORS-Tests (Lillif) and SHAPIRO-WILK-Test (SW)

Group	N	Std.Dev.	p KS	p Lillif	p SW
**stressed**	30	1.4559	>0.20	<0.05	= 0.0232
**control**	30	1.7006	>0.20	<0.05	= 0.0188

Comparison of the unpaired median data by the MANN-WHITNEY-U-Test (MannU) of the percentage of stops of the stressed and the control group.

Group	U	Z	2*1 sided exact p
**stressed vs. control**	296.00	-2.2768	0.0225

Tab. 1g refers to data presented in Figure 1g.

p-values of the KOLMOGOROV-SMIRNOV-Test (KS); LILLIFORS-Tests (Lillif) and SHAPIRO-WILK-Test (SW)

	**N**	**Std.Dev.**	**p KS**	**p Lillif**	**p SW**
**Pretest**	45	0.7804	< 0.01	< 0.01	$< 10^{-5}$
**Stress**	41	1.0927	< 0.20	< 0.01	= 0.0138
**Control**	41	0.9500	< 0.01	< 0.01	= 0.0002

Comparison of the unpaired median data by the MANN-WHITNEY-U-Test (MannU) of climbing attempts of males after courtship suppression and the control group

Group	(2)	(3)
K-Wallis-Test: H(2 N=127)=4.0942 p=0.1291		
**Pretest (1)**<br>R: 55.633	0.4257<br>1.4688	0.2191<br>1.7926
**stressed (2)**<br>R: 67.305	--	~1.0000<br>0.3165
**control (3)**<br>R: 69.878	--	--

Supplementary Table 2 | Statistical analysis for Figure 2.

Tab. 2a refers to data presented in Figure 2a.

p-values of the KOLMOGOROV-SMIRNOV-Test (KS); LILLIFORS-Tests (Lillif) and SHAPIRO-WILK-Test (SW)

	N	Std.Dev.	p KS	p Lillif	p SW
**Pretest** **_npf_-GAL4> UAS-_npf_^{RNAi}**	43	1.1191	< 0.15	< 0.01	= 0.0012
**Pretest** **_or83b_-GAL4> UAS-_npf_^{RNAi}**	43	1.1157	< 0.10	< 0.01	= 0.0016
**Test/Stress** **_npf_-GAL4> UAS-_npf_^{RNAi}**	42	1.1088	< 0.15	< 0.01	= 0.0091
**Test/Stress** **_or83b_-GAL4> UAS-_npf_^{RNAi}**	42	1.7539	> 0.20	< 0.10	= 0.3305
**Test/Control** **_npf_-GAL4> UAS-_npf_^{RNAi}**	43	1.3753	< 0.10	< 0.01	= 0.0269
**Test/Control** **_or83b_-GAL4> UAS-_npf_^{RNAi}**	42	1.4191	< 0.20	< 0.01	= 0.0741

Comparison of the unpaired median data by the KRUSKAL-WALLIS-TEST (K-W) of climbing attempts.
Upper row: multiple p-values; lower row: z-values

**Genotype**	**(2)**	**(3)**	**(4)**	**(5)**	**(6)**
K-Wallis-Test: H(5.N=255)=95.3927 p< 10^{-5}					
**Pretest** **_npf_-GAL4> UAS-_npf_ ^{RNAi} (1)** R: 169.91	~1.0000 0.1067	< 10^{-5} 7.2547	< 10^{-5} 4.9604	0.0988 2.7172	~1.0000 0.8312
**Pretest** **_or83b_-GAL4> UAS-_npf_ ^{RNAi} (2)** R: 50.655	--	< 10^{-5} 7.5588	< 10^{-5} 5.0665	0.0712 2.8239	~1.0000 0.9373
**Test(Stress)** **_npf_-GAL4> UAS-_npf_ ^{RNAi} (3)** R: 126.69	--	--	0.1983 2.4779	< 10^{-5} 4.7516	< 10^{-5} 6.5830
**Test(Stress)** **_or83b_-GAL4> UAS-_npf_ ^{RNAi} (4)** R: 171.60	--	--	--	0.3580 2.2592	0.0006 4.1051
**Test(Control)** **_npf_-GAL4> UAS-_npf_ ^{RNAi} (5)** R: 90.536	--	--	--	--	0.9224 1.8699
**Test(Control)** **_or83b_-GAL4> UAS-_npf_ ^{RNAi} (6)** R: 156.61	--	--	--	--	--

Tab. 2b refers to data presented in Figure 2b.

p-values of the KOLMOGOROV-SMIRNOV-Test (KS); LILLIFORS-Tests (Lillif) and SHAPIRO-WILK-Test (SW)

	N	Std.Dev.	p KS	p Lillif	p SW
**Pretest** **_npf_-GAL4> UAS-_npf_ ^{cDNA}**	17	1.0880	> 0.20	< 0.01	= 0.0064
**Pretest** **CS**	16	0.9309	< 0.15	< 0.01	= 0.0203
**Test** **_npf_-GAL4> UAS-_npf_ ^{cDNA}**	34	1.5786	> 0.20	< 0.05	= 0.1648
**Test** **CS**	31	1.6017	< 0.15	< 0.01	= 0.0635

Comparison of the unpaired median data by the KRUSKAL-WALLIS-TEST (K-W) of climbing attempts.
Upper row: multiple p-values; lower row: z-values

Group	(2)	(3)	(4)
K-Wallis-Test: H(3.N=98)=48.2537 $p < 10^{-5}$			
**Pretest/*npf*-GAL4> UAS-*npf* cDNA (1)**<br>R: 78.412	~1.0000<br>0.3129	$< 10^{-5}$<br>4.7428	$< 10^{-5}$<br>5.3445
**Pretest/CS (2)**<br>R: 38.353	--	0.0001<br>4.2875	$< 10^{-5}$<br>4.8858
**Test / *npf*-GAL4> UAS-*npf* cDNA (3)**<br>R: 75.313	--	--	~1.0000<br>0.8220
**Test/CS (4)**<br>R: 32.548	--	--	--

Tab. 2c refers to data presented in Figure 2c.

p-values of the KOLMOGOROV-SMIRNOV-Test (KS); LILLIFORS-Tests (Lillif) and SHAPIRO-WILK-Test (SW)

Group	N	Std.Dev.	p KS	p Lillif	p SW
**Pretest (*rut*[1])**	51	1.0167	< 0.01	< 0.01	= 0.0002
**Stress (*rut*[1])**	46	1.1583	< 0.10	< 0.01	= 0.0111
**Control (*rut*[1])**	42	1.2605	< 0.15	< 0.01	= 0.0155

Comparison of the unpaired median data by the KRUSKAL-WALLIS-TEST (K-W) of climbing attempts.
Upper row: multiple p-values; lower row: z-values

Group	(2)	(3)
K-Wallis-Test: H(2.N=139)=50.5958 $p < 10^{-5}$		
**Pretest (*rut*[1]) (1)**<br>R: 84.206	$< 10^{-5}$<br>5.8313	~1.0000<br>0.6295
**Stress (*rut*[1]) (2)**<br>R: 36.457	--	$< 10^{-5}$<br>6.1704
**Control (*rut*[1]) (3)**<br>R: 89.488	--	--

Tab. 2d refers to data presented in Figure 2d.

p-values of the KOLMOGOROV-SMIRNOV-Test (KS); LILLIFORS-Tests (Lillif) and SHAPIRO-WILK-Test (SW)

	N	Std.Dev.	p KS	p Lillif	p SW
**Pretest (*dnc*[1])**	49	1.1173	< 0.15	< 0.01	= 0.0024
**Stress (*dnc*[1])**	24	1.3805	> 0.20	< 0.15	= 0.1513
**Control (*dnc*[1])**	24	0.9237	< 0.10	< 0.01	= 0.0093

Comparison of the unpaired median data by the KRUSKAL-WALLIS-TEST (K-W) of climbing attempts.
Upper row: multiple p-values; lower row: z-values

Group	(2)	(3)
K-Wallis-Test: H(2.N=97)=51.3956 $p < 10^{-5}$		
**Pretest (*dnc*[1]) (1)**<br>R: 64.347	$< 10^{-5}$<br>7.0282	0.2076<br>0.6295
**Stress (*dnc*[1]) (2)**<br>R: 15.063	--	$< 10^{-5}$<br>4.4975
**Control (*dnc*[1]) (3)**<br>R: 51.604	--	--

Supplementary Table 3 | Statistical analysis for Figure 3.

Tab. 3a refers to data presented in Figure 3a.

p-values of the KOLMOGOROV-SMIRNOV-Test (KS); LILLIFORS-Tests (Lillif) and SHAPIRO-WILK-Test (SW)

	**N**	**Std.Dev.**	**p KS**	**p Lillif**	**p SW**
**Pretest stressed**	19	0.8377	< 0.15	< 0.01	= 0.0163
**Pretest control**	18	0.8556	< 0.20	< 0.10	= 0.0279
**Test 1 stressed**	17	0.8489	> 0.20	< 0.05	= 0.0332
**Test 1 control**	17	0.8703	< 0.15	< 0.01	= 0.0295
**Test 2 + 50 mM LiCl stressed**	14	1.0510	> 0.20	< 0.10	= 0.0516
**Test 2 + 50 mM LiCl control**	15	1.1339	> 0.20	< 0.05	= 0.0050

Comparison of the unpaired median data by the KRUSKAL-WALLIS-TEST (K-W) of climbing attempts.
Upper row: multiple p-values; lower row: z-values

	**(2)**	**(3)**	**(4)**	**(5)**	**(6)**
K-Wallis-Test: $H(5, N=100)= 73.6387$ $p< 10^{-5}$					
**Pretest (1) stressed** R: 42.00	~1.0000<br>0.3190	0.0061<br>3.5373	~1.0000<br>0.3922	0.0029<br>3.7254	0.0010<br>3.9995
**Pretest (2) control** R: 40.850	--	0.0022<br>3.8020	~1.0000<br>0.0769	0.0106<br>3.3878	0.0039<br>3.6513
**Test (3) stressed** R: 10.417	--	--	0.0020<br>3.8247	$< 10^{-5}$<br>6.9078	$< 10^{-5}$<br>7.2332
**Test (4) control** R: 33.150	--	--	--	0.0160<br>3.2729	0.0062<br>3.5300
**Test + 50mM LiCl (5) stressed** R: 70.038	--	--	--	--	~1.0000<br>0.1864
**Test + 50mM LiCl (6) control** R: 65.00	--	--	--	--	--

Tab. 3b refers to data presented in Figure 3b.

p-values of the KOLMOGOROV-SMIRNOV-Test (KS); LILLIFORS-Tests (Lillif) and SHAPIRO-WILK-Test (SW)

**Group**	**N**	**Std.Dev.**	**p KS**	**p Lillif**	**p SW**
**Pretest stressed**	21	0.8647	< 0.15	< 0.01	= 0.0041
**Pretest control**	21	0.8647	< 0.15	< 0.10	= 0.0279
**Test stressed**	21	1.1106	< 0.10	< 0.01	= 0.0137
**Test 1 control**	21	0.8136	< 0.10	< 0.01	= 0.0116
**Test 2 + 5 mM LiCL**	21	1.3887	> 0.20	> 0.15	= 0.0343

**stressed**					
**Test 2 + 5 mM LiCl control**	19	1.2402	> 0.20	< 0.05	= 0.0540

Comparison of the unpaired median data by the KRUSKAL-WALLIS-TEST (K-W) of climbing attempts.
Upper row: multiple p-values; lower row: z-values

Group	(2)	(3)	(4)	(5)	(6)
K-Wallis-Test: H(5 N=100)= 73.6387 $p< 10^{-5}$					
**Pretest (1) stressed** R: 42.00	~1.0000 $< 10^{-5}$	$< 10^{-5}$ 5.8755	~1.0000 1.0583	~1.0000 1.7903	~1.0000 0.6519
**Pretest (2) control** R: 40.850	--	$< 10^{-5}$ 5.8755	~1.0000 1.0583	~1.0000 1.7903	~1.0000 0.6519
**Test (3) stressed** R: 10.417	--	--	$< 10^{-5}$ 4.8172	0.0007 4.0852	$< 10^{-5}$ 5.0748
**Test (4) control** R: 33.150	--	--	--	~1.0000 0.7320	~1.0000 0.3796
**Test + 5mM LiCl (5) stressed** R: 70.038	--	--	--	--	~1.0000 1.0931
**Test + 5mM LiCl (6) control** R: 65.00	--	--	--	--	--

Supplementary Table 4 | Statistical analysis for Figure 4

Tab. 4a refers to data presented in Figure 4a.

p-values of the KOLMOGOROV-SMIRNOV-Test (KS); LILLIFORS-Tests (Lillif) and SHAPIRO-WILK-Test (SW)

	N	Std.Dev.	p KS	p Lillif	p SW
**Pretest**	125	1.0442	< 0.01	< 0.01	$< 10^{-5}$
**Test**	125	1.1555	< 0.01	< 0.01	$< 10^{-5}$
**Test 2 (H_2O)**	31	1.1188	< 0.20	< 0.05	= 0.0059
**Test 2 (5-HTP)**	30	1.5844	< 0.10	< 0.01	= 0.0426
**Test 2 (sucrose)**	30	1.4404	< 0.15	< 0.01	= 0.0061
**Test 2 (5-HTP & sucrose)**	30	1.4223	< 0.15	< 0.01	= 0.0081

Comparison of the unpaired median data by the KRUSKAL-WALLIS-TEST (K-W) of climbing attempts.
Upper row: multiple p-values; lower row: z-values

Group	(2)	(3)	(4)	(5)	(6)
K-Wallis-Test: H(5 N=371)= 229.9513 $p< 10^{-5}$					
**Pretest (1)**<br>R: 279.01	$< 10^{-5}$<br>13.4850	$< 10^{-5}$ 9.1319	0.0001<br>4.4548	0.1090<br>2.6844	~1.0000<br>1.3459
**Test (2)**<br>R: 96.084	--	~1.0000<br>0.6305	0.0012<br>3.9352	$< 10^{-5}$<br>5.7056	$< 10^{-5}$<br>7.0441
**Test 2 (H_2O) (3)**<br>R: 82.516	--	--	0.0045<br>3.6179	$< 10^{-5}$<br>5.0232	$< 10^{-5}$<br>6.0858
**Test 2 (5-HTP) (4)**<br>R: 181.88	--	--	--	~1.0000<br>1.3940	0.2155<br>2.4479
**Test 2 (sucrose) (5)**<br>R: 220.48	--	--	--	--	~1.0000<br>1.0539
**Test 2 (5-HTP+sucrose) (6)**<br>R: 249.67	--	--	--	--	--

Tab. 4b refers to data presented in Figure 4b.

p-values of the KOLMOGOROV-SMIRNOV-Test (KS); LILLIFORS-Tests (Lillif) and SHAPIRO-WILK-Test (SW)

	N	Std.Dev.	p KS	p Lillif	p SW
**Pretest**	105	1.1105	< 0.01	< 0.01	$< 10^{-5}$
**Test**	103	1.3691	< 0.01	< 0.01	= 0.0003
**Test 2 (H_2O)**	24	1.3486	< 0.10	< 0.01	= 0.0123
**Test 2 (sucrose)**	25	1.1180	< 0.15	< 0.01	= 0.0275
**Test 2 (αMTP)**	26	1.5616	< 0.20	< 0.01	= 0.0802
**Test 2 (αMTP+sucrose)**	26	1.0325	> 0.20	< 0.01	= 0.0295

Comparison of the unpaired median data by the KRUSKAL-WALLIS-TEST (K-W) of climbing attempts.
Upper row: multiple p-values; lower row: z-values.

Group	(2)	(3)	(4)	(5)	(6)
K-Wallis-Test: H(5 N=309)=208.8431 $p< 10^{-5}$					
**Pretest (1)**<br>R: 279.01	$< 10^{-5}$<br>11.2570	$< 10^{-5}$<br>8.6472	~1.0000<br>0.7840	$< 10^{-5}$<br>8.0892	$< 10^{-5}$<br>7.8190
**Test (2)**<br>R: 96.084	--	~1.0000<br>1.7441	$< 10^{-5}$<br>6.2195	~1.0000<br>0.9606	~1.0000<br>0.6909
**Test 2 (H_2O) (3)**<br>R: 82.516	--	--	$< 10^{-5}$<br>6.2356	~1.0000<br>0.6517	~1.0000<br>0.8608
**Test 2 (5-HTP) (4)**<br>R: 181.88	--	--	--	$< 10^{-5}$<br>5.7031	$< 10^{-5}$<br>5.4918
**Test 2 (sucrose) (5)**<br>R: 220.48	--	--	--	--	~1.0000<br>0.2431
**Test 2 (5-HTP+sucrose) (6)**<br>R: 249.67	--	--	--	--	--

Tab. 4c refers to data presented in Figure 4e.

p-values of the KOLMOGOROV-SMIRNOV-Test (KS); LILLIFORS-Tests (Lillif) and SHAPIRO-WILK-Test (SW).

Group	N	Std.Dev.	p KS	p Lillif	p SW
**stressed**	12	0.2386	> 0.20	< 0.05	= 0.1307
**control**	12	0.2441	> 0.20	> 0.20	= 0.6657

Comparison of the unpaired median data of the 5HT-quantification in the ellipsoid body by the MANN-WHITNEY-U-Test (MannU) of *Drosophila* males of the stressed and control group.

Group	U	Z	2*1 sided exact p
**stressed vs. control**	49.00	-1.3279	0.1978

Tab. 4d refers to data presented in Figure 4f.

p-values of the KOLMOGOROV-SMIRNOV-Test (KS); LILLIFORS-Tests (Lillif) and SHAPIRO-WILK-Test (SW).

Group	N	Std.Dev.	p KS	p Lillif	p SW
**stress**	12	0.4265	> 0.20	< 0.15	= 0.0632
**control**	13	0.1856	> 0.20	< 0.20	= 0.0653

Comparison of the normally distributed data of the 5HT-quantification in the α-lobe by the unpaired T-TEST of *Drosophila* males of the stressed and control group.

Group	T	F	p
**stressed vs. control**	2.3142	5.2771	0.0297

Comparison of the unpaired median data of the 5HT-quantification in the α-lobe by the MANN-WHITNEY-U-Test (MannU) of *Drosophila* males of the stressed and control group.

Group	U	Z	2*1 sided exact p
**stressed vs. control**	40.00	-2.0669	0.0398

Tab. 4e refers to data presented in Figure 4g.

p-values of the KOLMOGOROV-SMIRNOV-Test (KS); LILLIFORS-Tests (Lillif) and SHAPIRO-WILK-Test (SW).

Group	N	Std.Dev.	p KS	p Lillif	p SW
**stressed**	13	0.1717	> 0.20	> 0.20	= 0.8165
**control**	12	0.3861	> 0.20	> 0.20	= 0.8753

Comparison of the normally distributed data of the 5HT-quantification in the β-lobe by the unpaired T-TEST of *Drosophila* males of the stressed and control group.

Group	T	F	p
**stressed vs. control**	-1.1871	5.0543	0.2473

Comparison of the unpaired median data of the 5HT-quantification in the β -lobe by the MANN-WHITNEY-U-Test (MannU) of *Drosophila* males of the stressed and control group.

Group	U	Z	2*1 sided exact p
**stressed vs. control**	64.00	0.7615	0.4696

Tab. 4f refers to data presented in Figure 4h.

p-values of the KOLMOGOROV-SMIRNOV-Test (KS); LILLIFORS-Tests (Lillif) and SHAPIRO-WILK-Test (SW)

Group	N	Std.Dev.	p KS	p Lillif	p SW
**stressed**	13	0.1555	> 0.20	< 0.15	= 0.0040
**control**	12	0.4826	> 0.20	> 0.20	= 0.3733

Comparison of the unpaired median data of the 5HT-quantification in the γ-lobe by the MANN-WHITNEY-U-Test (MannU) of *Drosophila* males of the stressed and control group.

Group	U	Z	2*1 sided exact p
**stressed vs. control**	71.00	0.6923	0.5114

Supplementary Table 5 | Statistical analysis for Figure 5.

Tab. 5a refers to data presented in Figure 5a.

p-values of the KOLMOGOROV-SMIRNOV-Test (KS); LILLIFORS-Tests (Lillif) and SHAPIRO-WILK-Test (SW).

Group	N	Std.Dev.	p KS	p Lillif	p SW
**Pretest** **_Trh493_-GAL4> UAS-TNT**	23	0.8528	< 0.01	< 0.01	= 0.0003
**Pretest** **UAS-TNT/+**	25	0.8124	< 0.10	< 0.01	= 0.0026
**Test (stressed)** **_Trh493_-GAL4> UAS-TNT**	23	1.3112	> 0.20	< 0.05	= 0.1251
**Test (stressed)** **UAS-TNT/+**	24	1.1672	< 0.15	< 0.01	= 0.0038
**Test (sucrose)** **_Trh493_-GAL4> UAS-TNT**	23	1.2777	> 0.20	< 0.01	= 0.0310
**Test (sucrose)** **UAS-TNT/+**	25	0.8524	< 0.10	< 0.01	= 0.0060

Comparison of the unpaired median data by the KRUSKAL-WALLIS-TEST (K-W) of climbing attempts.
Upper row: multiple p-values; lower row: z-values.

Genotype	(2)	(3)	(4)	(5)	(6)
K-Wallis-Test: H(5 N=143)=103.5219 $p< 10^{-5}$					
**Pretest (1)** **_Trh493_-GAL4> UAS-TNT** R: 107.24	~1.0000 0.2374	$< 10^{-5}$ 5.7749	$< 10^{-5}$ 6.2262	$< 10^{-5}$ 5.3425	~1.0000 0.6032
**Pretest (2)** **UAS-TNT/+** R: 110.08	--	$< 10^{-5}$ 6.1314	$< 10^{-5}$ 6.5974	$< 10^{-5}$ 5.6900	~1.0000 0.8586
**Test (stressed) (3)** **_Trh493_-GAL4> UAS-TNT** R: 36.696	--	--	~1.0000 0.3902	~1.0000 0.4325	$< 10^{-5}$ 5.2908
**Test (stressed) (4)** **UAS-TNT/+** R: 31.979	--	--	--	~1.0000 0.8272	$< 10^{-5}$ 5.7476
**Test (sucrose) (5)** **_Trh493_-GAL4> UAS-TNT** R: 41.978	--	--	--	--	$< 10^{-5}$ 4.8495
**Test (sucrose) (6)** **UAS-TNT/+** R: 100.02	--	--	--	--	--

Tab. 5b refers to data presented in Figure 5b.

p-values of the KOLMOGOROV-SMIRNOV-Test (KS); LILLIFORS-Tests (Lillif) and SHAPIRO-WILK-Test (SW).

Group	N	Std.Dev.	p KS	p Lillif	p SW
**Pretest** ***Trh493*-GAL4> UAS-NaChBac**	45	0.7433	< 0.01	< 0.01	$< 10^{-5}$
**Pretest** **UAS-NaChBac/+**	44	0.9634	< 0.01	< 0.01	= 0.0005
**Test (stressed)** ***Trh493*-GAL4> UAS-NaChBac**	43	1.1206	< 0.05	< 0.01	= 0.0021
**Test (stressed)** **UAS-NaChBac/+**	44	1.2449	< 0.15	< 0.01	= 0.0212
**Test (sucrose)** ***Trh493*-GAL4> UAS-NaChBac**	20	0.8645	< 0.10	< 0.01	= 0.0055
**Test (sucrose)** **UAS-NaChBac/+**	22	1.1622	> 0.20	< 0.05	= 0.0622
**Test (sucrose+αMTP)** ***Trh493*-GAL4> UAS-NaChBac**	21	1.0282	> 0.20	< 0.05	= 0.0215
**Test (sucrose+αMTP)** **UAS-NaChBac/+**	21	0.9636	> 0.20	< 0.05	= 0.0531

Comparison of the unpaired median data by the KRUSKAL-WALLIS-TEST (K-W) of climbing attempts.
Upper row: multiple p-values; lower row: z-values.

Genotype	(2)	(3)	(4)	(5)	(6)	(7)	(8)
K-Wallis-Test: H(7 N=260)=152.0367 $p < 10^{-5}$							
**Pretest (1)** ***Trh493*-GAL4> UAS-NaChBac** R: 167.43	~1.0000 0.8737	~1.0000 1.0494	~1.0000 6.9851	~1.0000 0.1835	~1.0000 1.1466	$< 10^{-5}$ 5.7413	$< 10^{-5}$ 6.2661
**Pretest (2)** **UAS-NaChBac/+** R: 181.36	--	~1.0000 1.9075	$< 10^{-5}$ 7.8150	~1.0000 0.8698	~1.0000 0.4329	$< 10^{-5}$ 6.4191	$< 10^{-5}$ 6.9420
**Test (stress) (3)** ***Trh493*-GAL4> UAS-NaChBac** R: 150.60	--	--	$< 10^{-5}$ 5.8625	~1.0000 0.6446	~1.0000 1.9916	$< 10^{-5}$ 4.8587	$< 10^{-5}$ 5.3796
**Test (stress) (4)** **UAS-NaChBac/+** R: 56.068	--	--	--	$< 10^{-5}$ 5.3086	$< 10^{-5}$ 6.8138	~1.0000 0.1371	~1.0000 0.6600
**Test (sucrose) (5)** ***Trh493*-GAL4> UAS-NaChBac** R: 163.72	--	--	--	--	~1.0000 1.1250	0.0001 4.6984	$< 10^{-5}$ 5.1423
**Test (sucrose) (6)** **UAS-NaChBac/+** R: 189.86	--	--	--	--	--	$< 10^{-5}$ 5.9511	$< 10^{-5}$ 6.4057
**Test (sucrose+αMTP) (7)** ***Trh493*-GAL4> UAS-NaChBac** R: 53.33	--	--	--	--	--	--	~1.0000 0.4494
**Test (sucrose+αMTP) (8)** **UAS-NaChBac/+** R: 42.905	--	--	--	--	--	--	--

Tab. 5c refers to data presented in Figure 5f.

p-values of the KOLMOGOROV-SMIRNOV-Test (KS); LILLIFORS-Tests (Lillif) and SHAPIRO-WILK-Test (SW).

	N	Std.Dev.	p KS	p Lillif	p SW
**Pretest** ***mb247*-GAL4> UAS-TNT**	46	0.9631	< 0.05	< 0.01	= 0.0012
**Pretest** **UAS-TNT/+**	25	0.8124	< 0.10	< 0.01	= 0.0026
**Pretest** ***mb247*-GAL4/+**	23	0.9154	< 0.15	< 0.01	= 0.0107
**Test** ***mb247*-GAL4> UAS-TNT**	42	1.1527	< 0.01	< 0.01	= 0.0028
**Test** **UAS-TNT/+**	24	1.1672	< 0.15	< 0.01	= 0.0038
**Test** ***mb247*-GAL4/+**	23	1.5258	> 0.20	< 0.05	= 0.1750

Comparison of the unpaired median data by the KRUSKAL-WALLIS-TEST (K-W) of climbing attempts.
Upper row: multiple p-values; lower row: z-values.

**Genotype**	**(2)**	**(3)**	**(4)**	**(5)**	**(6)**
K-Wallis-Test: H(5 N=183)=92.5351 $p < 10^{-5}$					
**Pretest (1)** ***mb247*-GAL4> UAS-TNT** R: 122.83	0.8440<br>1.9090	~1.0000<br>0.1736	~1.0000<br>1.8148	$< 10^{-5}$<br>7.8870	$< 10^{-5}$<br>5.4011
**Pretest (2)** **UAS-TNT/+** R: 97.70	--	~1.0000<br>1.7951	~1.0000<br>0.3445	$< 10^{-5}$<br>5.2897	0.0260<br>3.1323
**Pretest (3)** **+/*mb247*-GAL4** R: 125.17	--	--	~1.0000<br>1.6640	$< 10^{-5}$<br>6.9580	$< 10^{-5}$<br>4.8278
**Test (4)** ***mb247*-GAL4> UAS-TNT** R: 102.31	--	--	--	$< 10^{-5}$<br>6.2477	0.0020<br>3.8243
**Test (5)** **UAS-TNT/+** R: 17.625	--	--	--	--	0.5642<br>2.0791
**Test (6)** **+/*mb247*-GAL4** R: 49.761	--	--	--	--	--

Tab. 5d refers to data presented in Figure 5g.

p-values of the KOLMOGOROV-SMIRNOV-Test (KS); LILLIFORS-Tests (Lillif) and SHAPIRO-WILK-Test (SW).

Group	N	Std.Dev.	p KS	p Lillif	p SW
**Pretest** ***H24*-GAL4> UAS-TNT**	22	0.9726	> 0.20	< 0.05	= 0.0097
**Pretest** **UAS-TNT/+**	25	0.8124	< 0.10	< 0.01	= 0.0026
**Pretest** **+/*H24*-GAL4**	22	0.7799	< 0.10	< 0.01	= 0.0059
**Test** ***H24*-GAL4> UAS-TNT**	22	1.2239	> 0.20	< 0.10	= 0.0290
**Test** **UAS-TNT/+**	24	1.1672	< 0.15	< 0.01	= 0.0038
**Test** **+/*H24*-GAL4**	22	0.2868	< 0.15	< 0.01	= 0.0548

Comparison of the unpaired median data by the KRUSKAL-WALLIS-TEST (K-W) of climbing attempts.
Upper row: multiple p-values; lower row: z-values.

Genotype	(2)	(3)	(4)	(5)	(6)
K-Wallis-Test: H(5 N=137)=83.3922 $p < 10^{-5}$					
**Pretest (1)**<br>***H24*-GAL4> UAS-TNT**<br>R: 94.636	~1.0000<br>0.3358	~1.0000<br>0.2906	~1.0000<br>1.6047	$< 10^{-5}$<br>6.3635	~1.0000<br>4.8596
**Pretest (2)**<br>**UAS-TNT/+**<br>R: 90.740	--	~1.0000<br>0.6355	~1.0000<br>1.3193	$< 10^{-5}$<br>6.2290	$< 10^{-5}$<br>4.6765
**Pretest (3)**<br>**+/*H24*-GAL4**<br>R: 98.114	--	--	0.8709<br>1.8952	$< 10^{-5}$<br>6.6603	$< 10^{-5}$<br>5.1502
**Test (4)**<br>***H24*-GAL4> UAS-TNT**<br>R: 75.432	--	--	--	$< 10^{-5}$<br>4.7243	0.0170<br>3.2550
**Test (5)**<br>**UAS-TNT/+**<br>R: 20.083	--	--	--	--	~1.0000<br>1.3993
**Test (6)**<br>**+/*H24*-GAL4**<br>R: 36.477	--	--	--	--	--

Tab. 5e refers to data presented in Figure 5h.

p-values of the KOLMOGOROV-SMIRNOV-Test (KS); LILLIFORS-Tests (Lillif) and SHAPIRO-WILK-Test (SW).

Group	N	Std.Dev.	p KS	p Lillif	p SW
**Pretest**<br>***c739*-GAL4> UAS-TNT**	21	1.1402	< 0.15	< 0.01	= 0.0220
**Pretest**<br>**UAS-TNT/+**	25	0.8124	< 0.10	< 0.01	= 0.0026
**Pretest**<br>**+/*c739*-GAL4**	21	0.8891	< 0.15	< 0.01	= 0.0074
**Test**<br>***c739-GAL4*> UAS-TNT**	21	1.1792	> 0.20	< 0.05	= 0.0850
**Test**<br>**UAS-TNT/+**	25	1.1672	< 0.15	< 0.01	= 0.0038
**Test**<br>**+/*c739*-GAL4**	21	0.9487	< 0.20	< 0.01	= 0.0712
**Test 2 (sucrose)**<br>***c739*-GAL4> UAS-TNT**	21	1.3002	> 0.20	< 0.05	= 0.0671
**Test 2 (sucrose)**<br>**UAS-TNT/+**	25	0.8524	< 0.10	< 0.01	= 0.0060
**Test 2 (sucrose)**<br>**+/*c739*-GAL4**	21	0.8647	< 0.15	< 0.01	= 0.0032

Comparison of the unpaired median data by the KRUSKAL-WALLIS-TEST (K-W) of climbing attempts.
Upper row: multiple p-values; lower row: z-values.

Genotype	(2)	(3)	(4)	(5)	(6)	(7)	(8)	(9)
K-Wallis-Test: H(8 N=200)=141.2200 $p < 10^{-5}$								
**Pretest (1)** ***c739*-GAL4> UAS-TNT** R: 146.10	~1.0000<br>0.3482	~1.0000<br>0.3292	$< 10^{-5}$<br>5.7504	$< 10^{-5}$<br>6.0430	$< 10^{-5}$<br>5.4892	$< 10^{-5}$<br>5.2279	~1.0000<br>0.4842	~1.0000<br>0.6718
**Pretest (2)** **UAS-TNT/+** R: 152.06	--	~1.0000<br>0.6914	$< 10^{-5}$<br>6.3434	$< 10^{-5}$<br>6.6792	$< 10^{-5}$<br>6.0710	$< 10^{-5}$<br>5.7986	~1.0000<br>0.8711	~1.0000<br>1.0486
**Pretest (3)** ***c739-GAL4*/+** R: 140.21	--	--	$< 10^{-5}$<br>5.4212	$< 10^{-5}$<br>5.7030	$< 10^{-5}$<br>5.1600	$< 10^{-5}$<br>4.8987	~1.0000<br>0.1409	~1.0000<br>0.3426
**Test (4)** ***c739*-GAL4> UAS-TNT** R: 43.381	--	--	--	~1.0000<br>0.1039	~1.0000<br>0.2613	~1.0000<br>0.5225	$< 10^{-5}$<br>5.5111	$< 10^{-5}$<br>5.0786
**Test (5)** **UAS-TNT/+** R: 41.583	--	--	--	--	~1.0000<br>0.3738	~1.0000<br>0.6436	$< 10^{-5}$<br>5.8171	$< 10^{-5}$<br>5.3491
**Test (6)** ***c739-GAL4*/+** R: 48.048	--	--	--	--	--	~1.0000<br>0.2613	$< 10^{-5}$<br>5.2387	$< 10^{-5}$<br>4.8174
**Test 2 (sucrose) (7)** ***c739*-GAL4> UAS-TNT** R: 52.714	--	--	--	--	--	--	$< 10^{-5}$<br>4.9663	0.0002<br>4.5561
**Test 2 (sucrose) (8)** **UAS-TNT/+** R: 137.80	--	--	--	--	--	--	--	~1.0000<br>0.2162
**Test 2 (sucrose) (9)** ***c739-GAL4*/+** R: 134.10	--	--	--	--	--	--	--	--

Supplementary Table 6 | Statistical analysis for Figure 6.

Tab. 6a refers to data presented in Figure 6a.

p-values of the KOLMOGOROV-SMIRNOV-Test (KS); LILLIFORS-Tests (Lillif) and SHAPIRO-WILK-Test (SW).

Group	N	Std.Dev.	p KS	p Lillif	p SW
**Pretest**<br>***mb247*-GAL4> UAS-5HT-1A^{RNAi}**	52	0.8042	< 0.01	< 0.01	$< 10^{-5}$
**Pretest**<br>**UAS-5HT-1A^{RNAi}/+**	52	0.7794	< 0.01	< 0.01	$< 10^{-5}$
**Pretest**<br>**+/*mb247*-GAL4**	52	0.8975	< 0.01	< 0.01	= 0.0001
**Test**<br>***mb247*-GAL4> UAS-5HT-1A^{RNAi}**	26	1.3633	> 0.20	< 0.10	= 0.1371
**Test**<br>**UAS-5HT-1A^{RNAi}/+**	26	1.2026	> 0.20	< 0.05	= 0.1197
**Test**<br>**+/*mb247*-GAL4**	26	1.5237	< 0.15	< 0.01	= 0.0795
**Test 2 (sucrose)**<br>***mb247*-GAL4> UAS-5HT-1A^{RNAi}**	26	1.2508	< 0.10	< 0.01	= 0.0645
**Test 2 (sucrose)**<br>**UAS-5HT-1A^{RNAi}/+**	24	1.1034	> 0.20	< 0.01	= 0.0185
**Test 2 (sucrose)**<br>**+/*mb247*-GAL4**	26	1.2102	< 0.10	< 0.01	= 0.0174

Comparison of the unpaired median data by the KRUSKAL-WALLIS-TEST (K-W) of climbing attempts.
Upper row: multiple p-values; lower row: z-values.

Genotype	(2)	(3)	(4)	(5)	(6)	(7)	(8)	(9)
K-Wallis-Test: H(8 N=310)=152.6476 $p < 10^{-5}$								
**Pretest (1)**<br>***mb247*-GAL4> 5HT1A^{RNAi}**<br>R: 187.84	~1.0000<br>0.2773	~1.0000<br>0.9354	$< 10^{-5}$<br>5.1991	$< 10^{-5}$<br>5.5073	$< 10^{-5}$<br>5.5555	$< 10^{-5}$<br>5.6475	~1.0000<br>1.3627	~1.0000<br>0.7284
**Pretest (2)**<br>**UAS-5HT1A^{RNAi}/+**<br>R: 192.71	--	~1.0000<br>0.6580	$< 10^{-5}$<br>5.4255	$< 10^{-5}$<br>5.7337	$< 10^{-5}$<br>5.7819	$< 10^{-5}$<br>5.8739	~1.0000<br>1.1423	~1.0000<br>0.5020
**Pretest (3)**<br>***mb247-GAL4*/+**<br>R: 204.28	--	--	$< 10^{-5}$<br>5.9628	$< 10^{-5}$<br>6.2710	$< 10^{-5}$<br>6.3193	$< 10^{-5}$<br>6.4112	~1.0000<br>0.6194	~1.0000<br>0.0353
**Test (4)**<br>***mb247*-GAL4> 5HT1A^{RNAi}**<br>R: 75.904	--	--	--	~1.0000<br>0.2669	~1.0000<br>0.3087	~1.0000<br>0.3883	$< 10^{-5}$<br>5.5996	$< 10^{-5}$<br>5.1334
**Test (5)**<br>**UAS-5HT1A^{RNAi}/+**<br>R: 69.269	--	--	--	--	~1.0000<br>0.0418	~1.0000<br>0.1215	$< 10^{-5}$<br>5.8611	$< 10^{-5}$<br>5.4003
**Test (6)**<br>***mb247-GAL4*/+**<br>R: 68.231	--	--	--	--	--	~1.0000<br>0.0797	$< 10^{-5}$<br>5.9020	$< 10^{-5}$<br>5.4420
**Test 2 (sucrose) (7)**<br>***mb247*-GAL4> 5HT1A^{RNAi}**<br>R: 66.250	--	--	--	--	--	--	$< 10^{-5}$<br>5.9801	$< 10^{-5}$<br>5.5217
**Test 2 (sucrose) (8)**<br>**UAS-5HT1A^{RNAi}/+**<br>R: 217.98	--	--	--	--	--	--	--	~1.0000<br>0.5699
**Test 2 (sucrose) (9)**<br>***mb247-GAL4*/+**<br>R: 203.52	--	--	--	--	--	--	--	--

Tab. 6b refers to data presented in Figure 6b.

p-values of the KOLMOGOROV-SMIRNOV-Test (KS); LILLIFORS-Tests (Lillif) and SHAPIRO-WILK-Test (SW).

Genotype	N	Std.Dev.	p KS	p Lillif	p SW
**Pretest** *mb247*-**GAL4> UAS-5HT-1B^{RNAi}**	50	0.7890	< 0.05	< 0.01	< 10^{-5}
**Pretest UAS-5HT-1B^{RNAi}/+**	50	0.7273	< 0.01	< 0.01	< 10^{-5}
**Pretest +/*mb247*-GAL4**	52	0.8975	< 0.01	< 0.01	= 0.0001
**Test** *mb247*-**GAL4> UAS-5HT-1B^{RNAi}**	24	1.0206	< 0.10	< 0.01	= 0.0206
**Test UAS-5HT-1B^{RNAi}/+**	26	1.2006	> 0.20	< 0.05	= 0.1340
**Test +/*mb247*-GAL4**	26	1.5237	< 0.15	< 0.01	= 0.0795
**Test 2 (sucrose)** *mb247*-**GAL4> UAS-5HT-1B^{RNAi}**	24	1.3270	> 0.20	< 0.15	= 0.1000
**Test 2 (sucrose) UAS-5HT-1B^{RNAi}/+**	25	1.0985	> 0.20	< 0.01	= 0.0075
**Test 2 (sucrose) +/*mb247*-GAL4**	26	1.2102	< 0.10	< 0.01	= 0.0174

Comparison of the unpaired median data by the KRUSKAL-WALLIS-TEST (K-W) of climbing attempts; data separated according to different days. Upper row: multiple p-values; lower row: z-values.

Genotype	(2)	(3)
K-Wallis-Test: H(2 N=152)=30.7643 p< 10^{-5}		
**Pretest (1)** *mb247*-**GAL4> UAS-5HT-1B^{RNAi}** R: 94.130	< 10^{-5} 4.9849	0.8535 1.0702
**Pretest (2) UAS-5HT-1B^{RNAi}/+** R: 50.240	--	0.0002 3.9633
**Pretest (3) +/*mb247*-GAL4** R: 84.798	--	--

Genotype	(2)	(3)
K-Wallis-Test: H(2 N=76)=28.0599 p< 10^{-5}		
**Test (1)** *mb247*-**GAL4> UAS-5HT-1B^{RNAi}** R: 56.146	< 10^{-5} 5.1855	0.0065 3.0659
**Test (2) UAS-5HT-1B^{RNAi}/+** R: 23.731	--	0.0915 2.1633
**Test (3) +/*mb247*-GAL4** R: 36.981	--	--

Genotype	(2)	(3)
K-Wallis-Test: H(2 N=75)=7.3992 p=0.0247		
**Test 2 (sucrose) (1)** *mb247*-**GAL4> UAS-5HT-1B^{RNAi}** R: 31.083	~1.0000 0.7220	0.0339 2.5332
**Test 2 (sucrose) (2) UAS-5HT-1B^{RNAi}/+** R: 35.580	--	0.2947 1.8234
**Test 2 (sucrose) (3) +/*mb247*-GAL4** R: 46.712	--	--

Tab. 6c refers to data presented in Figure 6c.

p-values of the KOLMOGOROV-SMIRNOV-Test (KS); LILLIFORS-Tests (Lillif) and SHAPIRO-WILK-Test (SW).

Genotype	N	Std.Dev.	p KS	p Lillif	p SW
**Pretest** **_H24_-GAL4> 5HT1A^{RNAi}**	25	0.8660	< 0.05	< 0.01	= 0.0037
**Pretest** **_c739_-GAL4> 5HT1A^{RNAi}**	25	0.8660	< 0.05	< 0.01	= 0.0037
**Test 1** **_H24_-GAL4> 5HT1A^{RNAi}** **Stressed**	23	0.9472	< 0.10	< 0.01	= 0.0077
**Test 1** **_c739_-GAL4> 5HT1A^{RNAi}** **Stressed**	25	1.0924	< 0.10	< 0.01	= 0.0095
**Test 2 (Sucrose)** **_H24_-GAL4> 5HT1A^{RNAi}** **Stressed**	20	0.8256	< 0.15	< 0.01	= 0.0145
**Test 2 (Sucrose)** **_c739_-GAL4>5HT1A^{RNAi}** **Stressed**	25	1.2083	> 0.20	< 0.01	= 0.0371

Comparison of the unpaired median data by the KRUSKAL-WALLIS-TEST (K-W).
Upper row: multiple p-values; lower row: z-values.

Genotyp	(2)	(3)	(4)	(5)	(6)
K-Wallis-Test: $H(5\ N=143)= 106.6746\ p< 10^{-5}$					
**Pretest (1)** **_H24_-GAL4> 5HT1A^{RNAi}** R: 105.92	~1.0000 < 10^{-5}	< 10^{-5} 5.0427	< 10^{-5} 5.8259	~1.0000 0.5094	< 10^{-5} 6.4267
**Pretest (2)** **_c739_-GAL4> 5HT1A^{RNAi}** R: 105.92	--	< 10^{-5} 5.0427	< 10^{-5} 5.8259	~1.0000 0.5094	< 10^{-5} 6.4267
**Test 1/Stressed (3)** **_H24_-GAL4> 5HT1A^{RNAi}** R: 45.565	--	--	~1.0000 0.6605	< 10^{-5} 5.2652	~1.0000 1.2487
**Test 1/Stressed (4)** **_c739_-GAL4> 5HT1A^{RNAi}** R: 37.660	--	--	--	< 10^{-5} 6.0021	~1.0000 0.6009
**Test 2/Stressed (Sucrose)(5)** **_H24_-GAL4> 5HT1A^{RNAi}** R: 112.25	--	--	--	--	< 10^{-5} 6.5686
**Test 2/Stressed (Sucrose) (6)** **_c729_-GAL4> 5HT1A^{RNAi}** R: 30.620	--	--	--	--	--

Tab. 6d refers to data presented in Figure 6d.

p-values of the KOLMOGOROV-SMIRNOV-Test (KS); LILLIFORS-Tests (Lillif) and SHAPIRO-WILK-Test (SW).

Genotype	N	Std.Dev.	p KS	p Lillif	p SW
**Pretest**<br>***H24*-GAL4> 5HT1B^{RNAi}**	26	1.0266	< 0.05	< 0.01	= 0.0137
**Pretest**<br>***c739*-GAL4> 5HT1B^{RNAi}**	26	1.0077	< 0.15	< 0.01	= 0.0026
**Test 1**<br>***H24*-GAL4> 5HT1B^{RNAi}**<br>**Stressed**	20	0.9403	< 0.10	< 0.01	= 0.0148
**Test 1**<br>***c739*-GAL4> 5HT1B^{RNAi}**<br>**Stressed**	21	1.3381	> 0.20	< 0.05	= 0.0793

Comparison of the unpaired median data by the KRUSKAL-WALLIS-TEST (K-W).
Upper row: multiple p-values; lower row: z-values.

Genotyp	(2)	(3)	(4)
K-Wallis-Test: H(3 N=93)= 47.2488 p< 10^{-5}			
**Pretest (1)**<br>***H24*-GAL4> 5HT1B^{RNAi}**<br>R: 58.404	~1.0000<br>0.8580	~1.0000<br>0.4075	< 10^{-5}<br>5.7666
**Pretest (2)**<br>***c739*-GAL4> 5HT1B^{RNAi}**<br>R: 51.981	--	~1.0000<br>1.2076	< 10^{-5}<br>4.9555
**Test/Stressed (3)**<br>***H24*-GAL4> 5HT1B^{RNAi}**<br>R: 61.675	--	--	< 10^{-5}<br>5.8030
**Test/Stress (4)**<br>***c739*-GAL4> 5HT1B^{RNAi}**<br>R: 12.738	--	--	--

Supplementary Table 7 | Statistical analysis for Figure 7.

Tab. 7a refers to data presented in Figure 7a.

p-values of the KOLMOGOROV-SMIRNOV-Test (KS); LILLIFORS-Tests (Lillif) and SHAPIRO-WILK-Test (SW).

Genotype	N	Std.Dev.	p KS	p Lillif	p SW
**Pretest / *5HT-1A*-GAL4>UAS-NaChBac**	26	0.8038	< 0.01	< 0.01	$< 10^{-5}$
**Pretest / UAS-NaChBac/+**	21	0.8891	< 0.05	< 0.01	= 0.0003
**Pretest / +/*5HT-1A*-GAL4**	20	0.9787	> 0.20	< 0.05	= 0.0464
**Test / *5HT-1A*-GAL4>UAS-NaChBac**	26	1.0763	< 0.15	< 0.01	= 0.0009
**Test / UAS-NaChBac/+**	21	1.2611	> 0.20	< 0.05	= 0.1032
**Test / +/*5HT-1A*-GAL4**	19	1.1161	< 0.15	< 0.01	= 0.0271

Comparison of the unpaired median data by the KRUSKAL-WALLIS-TEST (K-W) of climbing attempts.
Upper row: multiple p-values; lower row: z-values.

Genotype	(2)	(3)	(4)	(5)	(6)
K-Wallis-Test: H(5 N=133)=72.9694 $p< 10^{-5}$					
**Pretest (1)**<br>***5HT-1A*-GAL4> UAS-NaChBac** R: 78.923	~1.0000<br>0.8175	~1.0000<br>0.3382	~1.0000<br>1.0579	$< 10^{-5}$<br>4.9017	~1.0000<br>4.4686
**Pretest (2)**<br>**UAS- NaChBac /+** R: 88.167	--	~1.0000<br>0.4457	~1.0000<br>0.1826	$< 10^{-5}$<br>5.4374	$< 10^{-5}$<br>5.0172
**Pretest (3)**<br>**+/*5HT-1A*-GAL4** R: 82.800	--	--	~1.0000<br>0.6483	$< 10^{-5}$<br>4.9249	0.0001<br>4.5239
**Test (4)**<br>***5HT-1A*-GAL4> UAS-NaChBac** R: 90.231	--	--	--	$< 10^{-5}$<br>5.9018	$< 10^{-5}$<br>5.4408
**Test (5)**<br>**UAS-NaChBac /+** R: 23.50	--	--	--	--	~1.0000<br>0.2825
**Test (6)**<br>**+/*5HT-1A*-GAL4** R: 26.947	--	--	--	--	--

Tab. 7b refers to data presented in Figure 7b.

p-values of the KOLMOGOROV-SMIRNOV-Test (KS); LILLIFORS-Tests (Lillif) and SHAPIRO-WILK-Test (SW).

Genotype	N	Std.Dev.	p KS	p Lillif	p SW
**Pretest**<br>***5HT-1B*-GAL4> UAS-NaChBac**	63	0.8957	< 0.01	< 0.01	$< 10^{-5}$
**Pretest**<br>**UAS-NaChBac/+**	64	0.8858	< 0.01	< 0.01	$< 10^{-5}$
**Pretest**<br>**+/*5HT-1B*-GAL4**	60	0.7702	< 0.01	< 0.01	$< 10^{-5}$
**Test 1**<br>***5HT-1B*-GAL4> UAS-NaChBac**	21	1.5675	< 0.20	< 0.01	= 0.0924
**Test 1**<br>**UAS-NaChBac/+**	23	0.7777	< 0.05	< 0.01	= 0.0035
**Test 1**<br>**+/*5HT-1B*-GAL4**	20	0.9881	< 0.15	< 0.01	= 0.0436
**Test 2**<br>***5HT-1B*-GAL4> UAS-NaChBac**	20	1.0311	< 0.15	< 0.01	= 0.0056
**Test 2**<br>**UAS-NaChBac/+**	21	1.1233	> 0.20	< 0.01	= 0.0336
**Test 2**<br>**+/*5HT-1B*-GAL4**	19	1.0976	> 0.20	< 0.10	= 0.0114
**Test 3**<br>***5HT-1B*-GAL4> UAS-NaChBac**	20	0.9403	< 0.05	< 0.01	= 0.0022
**Test 3**<br>**UAS-NaChBac/+**	20	1.0311	< 0.10	< 0.01	= 0.0114
**Test 3**<br>**+/*5HT-1B*-GAL4**	19	1.6490	< 0.20	< 0.01	= 0.1887

Genotype	(2)	(3)	(4)	(5)	(6)	(7)	(8)	(9)	(10)	(11)	(12)
K-Wallis-Test: H(11 N=370)=199.0453 $p< 10^{-5}$											
**Pretest (1)**<br>***5HT-1B*-GAL4>NaChBac**<br>R: 218.95	~1.0000<br>2.2699	~1.0000<br>0.2500	0.0003<br>4.5905	~1.0000<br>1.5812	~1.0000<br>0.8088	$< 10^{-5}$<br>6.4990	~1.0000<br>0.1793	~1.0000<br>1.7290	$< 10^{-5}$<br>6.3752	$< 10^{-5}$<br>5.1311	0.0001<br>4.9384
**Pretest (2)**<br>**UAS-NaChBac/+**<br>R: 262.04	--	~1.0000<br>1.9909	$< 10^{-5}$<br>6.2014	~1.0000<br>0.0726	~1.0000<br>2.3829	$< 10^{-5}$<br>8.0839	~1.0000<br>1.4222	0.0670<br>3.2741	$< 10^{-5}$<br>7.9598	$< 10^{-5}$<br>6.7134	$< 10^{-5}$<br>6.4893
**Pretest (3)**<br>**+/*5HT-1B*-GAL4**<br>R: 223.78	--	--	0.0001<br>4.7399	~1.0000<br>1.3869	~1.0000<br>0.9786	$< 10^{-5}$<br>6.6349	~1.0000<br>0.0004	~1.0000<br>1.8903	$< 10^{-5}$<br>6.5118	$< 10^{-5}$<br>5.2751	$< 10^{-5}$<br>5.0813
**Test 1 (4)**<br>***5HT-1B*-GAL4>NaChBac**<br>R: 95.238	--	--	--	$< 10^{-5}$<br>5.1087	0.1573<br>3.0378	~1.0000<br>1.6365	0.0065<br>3.8946	~1.0000<br>2.2240	~1.0000<br>1.5348	~1.0000<br>0.5129	~1.0000<br>0.4290
**Test 1 (5)**<br>**UAS-NaChBac/+**<br>R: 260.15	--	--	--	--	~1.0000<br>1.9389	$< 10^{-5}$<br>6.7156	~1.0000<br>1.1266	0.4546<br>2.7022	$< 10^{-5}$<br>6.6116	$< 10^{-5}$<br>5.5673	$< 10^{-5}$<br>5.4118
**Test 1 (6)**<br>**+/*5HT-1B*-GAL4**<br>R: 196.75	--	--	--	--	--	0.0003<br>4.6183	~1.0000<br>0.8090	~1.0000<br>0.7646	0.0004<br>4.5178	0.0298<br>3.5081	0.0467<br>3.3866
**Test 2 (7)**<br>***5HT-1B*-GAL4>NaChBac**<br>R: 40.550	--	--	--	--	--	--	$< 10^{-5}$<br>5.4833	0.0098<br>3.7942	~1.0000<br>0.1005	~1.0000<br>1.1102	~1.0000<br>1.1721
**Test 2 (8)**<br>**UAS-NaChBac/+**<br>R: 223.79	--	--	--	--	--	--	--	~1.0000<br>1.5720	$< 10^{-5}$<br>5.3816	0.0009<br>4.3597	0.0016<br>4.2250
**Test 2 (9)**<br>**+/*5HT-1B*-GAL4**<br>R: 170.55	--	--	--	--	--	--	--	--	0.0145<br>3.6949	0.4601<br>2.6982	0.6351<br>2.5891
**Test 3 (7)**<br>***5HT-1B*-GAL4>NaChBac**<br>R: 43.950	--	--	--	--	--	--	--	--	--	~1.0000<br>1.0097	~1.0000<br>1.0729
**Test 3 (8)**<br>**UAS-NaChBac/+**<br>R: 78.100	--	--	--	--	--	--	--	--	--	--	~1.0000<br>0.0762
**Test 3 (9)**<br>**+/*5HT-1B*-GAL4**<br>R: 80.711	--	--	--	--	--	--	--	--	--	--	--

Tab. 7c refers to data presented in Figure 7c.

p-values of the KOLMOGOROV-SMIRNOV-Test (KS); LILLIFORS-Tests (Lillif) and SHAPIRO-WILK-Test (SW).

	**N**	**Std.Dev.**	**p KS**	**p Lillif**	**p SW**
**Pretest Group 1 (CS)**	21	0.9258	< 0.10	< 0.01	= 0.0109
**Pretest Group 2 (CS)**	21	0.9437	< 0.20	< 0.01	= 0.0265
**Test 1/Stressed Group 1 (CS)**	21	1.3274	> 0.20	< 0.10	= 0.2061
**Test 1/Stressed Group 2 (CS)**	21	1.1792	> 0.20	< 0.05	= 0.0581
**Test 2/Stressed Group 1 + SSRI**	18	1.1448	> 0.20	< 0.05	= 0.1330
**Test 2/Stressed Group 2 + H_2O**	21	1.2440	> 0.20	< 0.05	= 0.2987

Comparison of the unpaired median data by the KRUSKAL-WALLIS-TEST (K-W).
Upper row: multiple p-values; lower row: z-values.

	**(2)**	**(3)**	**(4)**	**(5)**	**(6)**
K-Wallis-Test: H(5 N=123)= 80.0898 p< 10^{-5}					
**Pretest (1)** **Group 1 (CS)** R: 95.095	~1.0000 0.3051	< 10^{-5} 5.5898	< 10^{-5} 5.2717	~1.0000 1.7815	< 10^{-5} 5.4729
**Pretest 1 (2)** **Group 2 (CS)** R: 98.452	--	< 10^{-5} 5.8949	< 10^{-5} 5.5768	0.5703 2.0747	< 10^{-5} 5.7781
**Test 1/Stressed (3)** **Group 1 (CS)** R: 33.595	--	--	~1.0000 0.3181	0.0050 3.5890	~1.0000 0.1169
**Test 1/Stressed (4)** **Group 2 (CS)** R: 37.095	--	--	--	0.0154 3.2834	~1.0000 0.2013
**Test 2/Stressed (5)** **Group 1 + Prozac** R: 74.694	--	--	--	--	0.0076 3.4767
**Test 2/Stressed (6)** **Group 2 + H_2O** R: 34.881	--	--	--	--	--

Tab. 7d refers to data presented in Figure 7d.

p-values of the KOLMOGOROV-SMIRNOV-Test (KS); LILLIFORS-Tests (Lillif) and SHAPIRO-WILK-Test (SW).

Genotype	N	Std.Dev.	p KS	p Lillif	p SW
**Pretest**<br>***H24*-GAL4>UAS-TNT**	25	0.7024	< 0.10	< 0.01	= 0.0003
**Test 1**<br>***H24*-GAL4>UAS-TNT+8-OH-DPAT**	24	1.0206	> 0.20	< 0.05	= 0.0095
**Pretest**<br>**UAS-TNT/II**	25	1.2069	< 0.10	< 0.01	= 0.0280
**Test 1**<br>**UAS-TNT/II+8-OH-DPAT**	25	1.1662	< 0.20	< 0.01	= 0.0194
**Pretest**<br>***H24*-GAL4/II**	25	0.9183	< 0.15	< 0.01	= 0.0077
**Test 1**<br>***H24*-GAL4/II+8-OH-DPAT**	24	1.1034	> 0.20	< 0.10	= 0.0346

Comparison of the unpaired median data by the KRUSKAL-WALLIS-TEST (K-W).
Upper row: multiple p-values; lower row: z-values.

Genotyp	(2)	(3)	(4)	(5)	(6)
K-Wallis-Test: H(5 N=148)= 80.5844 $p< 10^{-5}$					
**Pretest (1)**<br>***H24*-GAL4>UAS-TNT**<br>R: 110.84	~1.0000<br>1.3066	0.0908<br>2.7448	< 10^{-5}<br>6.9048	~1.0000<br>1.1464	< 10^{-5}<br>5.8557
**Test 1**<br>***H24*-GAL4>UAS-TNT+8-OH-DPAT**<br>R: 94.833	--	~1.0000<br>1.4100	< 10^{-5}<br>5.5274	~1.0000<br>0.1720	0.0001<br>4.5034
**Pretest**<br>**UAS-TNT/II**<br>R: 77.560	--	--	0.0005<br>4.1600	~1.0000<br>1.5984	0.0254<br>3.1391
**Test 1**<br>**UAS-TNT/II+8-OH-DPAT**<br>R: 27.120	--	--	--	< 10^{-5}<br>5.7584	~1.0000<br>0.9783
**Pretest**<br>***H24*-GAL4/II**<br>R: 96.940	--	--	--	--	< 10^{-5}<br>4.7211
**Test 1**<br>***H24*-GAL4/II+8-OH-DPAT**<br>R: 39.104	--	--	--	--	--

Supplementary Table 8 | Statistical analysis for Figure S1.

Tab. 8a refers to data presented in Figure S1a.

p-values of the KOLMOGOROV-SMIRNOV-Test (KS); LILLIFORS-Tests (Lillif) and SHAPIRO-WILK-Test (SW).

	N	Std.Dev.	p KS	p Lillif	p SW
**CS no object**	29	0.9285	< 0.10	< 0.01	= 0.0130
**CS vis. obj.**	28	0.7935	< 0.05	< 0.01	= 0.0014
**CS odour**	28	0.9201	< 0.05	< 0.01	= 0.0036
**CS vis. obj. + odour**	28	0.8698	< 0.10	< 0.01	= 0.0028

Comparison of the unpaired median data by the KRUSKAL-WALLIS-TEST (K-W) of climbing attempts.
Upper row: multiple p-values; lower row: z-values.

	(2)	(3)	(4)
K-Wallis-Test: H(3,N=113)=73,7944 p< 10^{-5}			
**CS no obj. (1)**<br>R: 111.93	< 10^{-5}<br>5.2158	< 10^{-5}<br>4.9710	< 10^{-5}<br>8.2809
**CS vis. obj. (2)**<br>R: 118.20	--	~1,0000<br>0.2427	0.0143<br>3.0385
**CS odour (3)**<br>R: 102.28	--	--	0.0062<br>3.2812
**CS vis. obj. + odour (4)**<br>R: 104.70	--	--	--

Tab. 8b refers to data presented in Figure S1b.

p-values of the KOLMOGOROV-SMIRNOV-Test (KS); LILLIFORS-Tests (Lillif) and SHAPIRO-WILK-Test (SW).

Genotype	N	Std.Dev.	p KS	p Lillif	p SW
**CS 1st run no stimulus (Group I)**	24	1.5598	> 0.20	< 0.15	= 0.0941
**CS 1st run no stimulus (Group II)**	23	1.5616	> 0.20	< 0.10	= 0.2649
**CS 2nd run (24h later) odour**	24	1.0734	< 0.10	< 0.01	= 0.0061
**CS 2nd run (24h later) no odour**	23	1.4904	< 0.10	< 0.01	= 0.0043
***H24*>TNT 1st run no stimulus (Group I)**	27	1.5021	> 0.20	< 0.10	= 0.1796
***H24*>TNT 1st run no stimulus (Group II)**	22	1.7563	> 0.20	< 0.01	= 0.1867
***H24*>TNT 2nd run (24h later) odour**	27	1.6486	> 0.20	< 0.05	= 0.0876
***H24*>TNT 2nd run (24h later) no odour**	22	1.3266	< 0.20	< 0.01	= 0.0261
***c305a*>TNT 1st run no stimulus (Group I)**	23	1.1995	> 0.20	< 0.01	= 0.0208
***c305a*>TNT 1st run no stimulus (Group II)**	24	1.2959	> 0.20	< 0.01	= 0.0163
***c305a*>TNT 2nd run (24h later) odour**	23	1.3817	> 0.20	< 0.15	= 0.0335
***c305a*>TNT 2nd run (24h later) no odour**	24	1.2504	> 0.20	< 0.05	= 0.0827
***mb247*>TNT 1st run no stimulus (Group I)**	21	1.8860	> 0.20	< 0.05	= 0.0588
***mb247*>TNT 1st run no stimulus (Group II)**	20	1.4681	> 0.20	> 0.20	= 0.1738
***mb247*>TNT 2nd run (24h later) odour**	21	1.8297	> 0.20	< 0.15	= 0.1487
***mb247*>TNT 2nd run (24h later) no odour**	20	1.5044	> 0.20	< 0.05	= 0.1704
***c739*>TNT 1st run no stimulus (Group I)**	21	1.3990	> 0.20	< 0.05	= 0.1623
***c739*>TNT 1st run no stimulus (Group II)**	21	1.1019	< 0.20	< 0.01	= 0.0248
***c739*>TNT 2nd run (24h later) odour**	21	1.8746	> 0.20	< 0.10	= 0.1290
***c739*>TNT 2nd run (24h later) no odour**	21	1.8949	> 0.20	< 0.10	= 0.1100

Comparison of the paired data of the climbing behaviour of *Drosophila* males by the WILCOXON-MATCHED-PAIRS Test of their first run without odour and their second run paired with odour.

Genotype	N	T	Z	p
**CS 2nd run (24 h) + *odour*** **vs.** **CS *1st run - no stimulus (Group I)***	24	11.50	3.4906	0.0005
H24*>TNT 2nd run (24 h) + *odour **vs.** ***H24*>TNT *1st run - no stimulus (Group I)***	27	32.00	3.0680	0.0022
c305a*>TNT 2nd run (24 h) + *odour **vs.** ***c305a*>TNT *1st run - no stimulus (Group I)***	23	12.00	3.5974	0.0003
mb247*>TNT 2nd run (24 h) + *odour **vs.** ***mb247*>TNT *1st run - no stimulus (Group I)***	21	56.00	0.2272	0.8203
**c739>TNT 2nd run (24 h) + *odour*** **vs.** **c739>TNT *1st run - no stimulus (Group I)***	21	60.50	0.3878	0.6982

Comparison of the paired data of the climbing behaviour of *Drosophila* males by the WILCOXON-MATCHED-PAIRS Test of their first run without odour and their second run without odour.

Genotype	N	T	Z	p
**CS *1st run - no stimulus (Group II)*** **vs.** **CS 2nd run (24 h) *no odour***	23	21.00	2.6273	0.0086
H24*>TNT *1st run - no stimulus (Group II) **vs.** ***H24*>TNT 2nd run (24 h) *no odour***	22	84.00	0.7840	0.4330
c305a*>TNT *1st run - no stimulus (Group II) **vs.** ***c305a*>TNT 2nd run (24 h) *no odour***	24	88.00	0.2817	0.7782
mb247*>TNT *1st run - no stimulus (Group II) **vs.** ***mb247*>TNT 2nd run (24 h) *no odour***	20	48.00	1.3491	0.1773
c739*>TNT *1st run - no stimulus (Group II) **vs.** **c739>TNT 2nd run (24 h) *no odour***	21	35.50	1.3915	0.1641

Comparison of the unpaired data of the climbing behaviour of *Drosophila* males by the MANN-WHITNEY-U-Test (Mann U) of their second run with a food-odour and their second run without an odour.

Group	U	Z	2*1 sided exact p
**CS 2nd run (24 h) + *odour*** **vs.** **CS 2nd run (24 h) +*no odour***	142.50	2.8411	0.0038
H24*>TNT 2nd run (24 h) + *odour **vs.** ***H24*>TNT 2nd run (24 h) +*no odour***	232.50	1.2965	0.1967
c305a*>TNT 2nd run (24 h) + *odour **vs.** ***c305a*>TNT 2nd run (24 h) + *no odour***	187.50	1.8834	0.0592
mb247*>TNT 2nd run (24 h) + *odour **vs.** ***mb247*>TNT 2nd run (24 h) + *no odour***	207.50	-0.0652	0.9486
c739*>TNT 2nd run (24 h) + *odour **vs.** ***c739*>TNT 2nd run (24 h) + *no odour***	193.00	0.6918	0.5010

Tab. 8c refers to data presented in Figure S1c.

p-values of the KOLMOGOROV-SMIRNOV-Test (KS); LILLIFORS-Tests (Lillif) and SHAPIRO-WILK-Test (SW).

Genotype	N	Std.Dev.	p KS	p Lillif	p SW
**CS 1st run no stimulus (Group I)**	25	0.9363	< 0.10	< 0.01	= 0.0051
**CS 1st run no stimulus (Group II)**	25	1.2356	< 0.20	< 0.01	= 0.0252
**CS 2nd run (24h later) vis. obj.**	25	1.1136	> 0.20	< 0.05	= 0.0032
**CS 2nd run (24h later) no obj.**	25	1.3229	< 0.15	< 0.01	= 0.0670
***H24*>TNT 1st run no stimulus (Group I)**	17	0.9034	< 0.10	< 0.01	= 0.0106
***H24*>TNT 1st run no stimulus (Group II)**	18	0.9003	< 0.05	< 0.01	= 0.0028
***H24*>TNT 2nd run (24h later) vis. obj.**	17	1.0146	> 0.20	< 0.05	= 0.0252
***H24*>TNT 2nd run (24h later) no obj.**	18	0.8264	> 0.20	< 0.01	= 0.0234
***NP1131*>TNT 1st run no stimulus (Group I)**	23	0.9679	> 0.20	< 0.01	= 0.0311
***NP1131*>TNT 1st run no stimulus (Group II)**	23	0.9960	< 0.20	< 0.01	= 0.0278
***NP1131*>TNT 2nd run (24h later) vis. obj.**	23	1.2175	> 0.20	< 0.05	= 0.0524
***NP1131*>TNT 2nd run (24h later) no obj.**	23	1.3644	> 0.20	< 0.15	= 0.3714
***mb247*>TNT 1st run no stimulus (Group I)**	15	0.8619	> 0.20	< 0.01	= 0.0502
***mb247*>TNT 1st run no stimulus (Group II)**	15	1.1255	< 0.20	< 0.01	= 0.0568
***mb247*>TNT 2nd run (24h later) vis. obj.**	15	1.0556	< 0.10	< 0.01	= 0.0149
***mb247*>TNT 2nd run (24h later) no obj.**	15	0.8619	< 0.20	< 0.01	= 0.0028
***c739*>TNT 1st run no stimulus (Group I)**	20	1.5183	> 0.20	< 0.05	= 0.1106
***c739*>TNT 1st run no stimulus (Group II)**	20	1.1821	< 0.20	< 0.01	= 0.0429
***c739*>TNT 2nd run (24h later) vis. obj.**	20	1.5009	> 0.20	< 0.10	= 0.1099
***c739*>TNT 2nd run (24h later) no obj.**	20	1.6376	> 0.20	> 0.20	= 0.5225

Comparison of the paired data of the climbing behaviour of *Drosophila* males by the WILCOXON-MATCHED-PAIRS Test of their first run without visual object and their second run with a visual object.

Genotype	N	T	Z	p
**CS 2nd run (24 h) + *vis.obj.* vs. CS *1st run - no stimulus (Group I)***	25	0.00	4.1069	$<10^{-5}$
H24*>TNT 2nd run (24 h) + *vis.obj.* vs. *H24*>TNT *1st run - no stimulus (Group I)	17	0.00	3.4078	0.0007
NP1131*>TNT 2nd run (24 h) + *vis.obj.* vs. *NP1131*>TNT *1st run - no stimulus (Group I)	23	23.00	3.2151	0.0013
mb247*>TNT 2nd run (24 h) + *vis.obj.* vs. *mb247*>TNT *1st run - no stimulus (Group I)	15	13.00	1.1255	0.2604
c739*>TNT 2nd run (24 h) + *vis.obj.* vs. *c739*>TNT *1st run - no stimulus (Group I)	20	42.50	1.3186	0.1873

Comparison of the paired data of the climbing behaviour of *Drosophila* males by the WILCOXON-MATCHED-PAIRS Test of their first run without visual object and their second run also without visual object.

Genotype	N	T	Z	p
**CS *1st run - no stimulus (Group II)* vs. CS 2nd run (24 h) *no vis.obj.***	25	73.50	0.5226	0.6013
H24*>TNT *1st run - no stimulus (Group II)* vs. *H24*>TNT 2nd run (24 h) *no vis.obj.	18	33.00	0.4707	0.6379
c305a*>TNT *1st run - no stimulus (Group II)* vs. *c305a*>TNT 2nd run (24 h) *no vis.obj.	23	51.00	0.0942	0.9250
mb247*>TNT *1st run - no stimulus (Group II)* vs. *mb247*>TNT 2nd run (24 h) *no vis.obj.	15	39.00	0.8475	0.3967
**c739>TNT *1st run - no stimulus (Group II)* vs. c739>TNT 2nd run (24 h) *no vis.obj.***	20	49.50	0.9566	0.3388

Comparison of the unpaired data of the climbing behaviour of *Drosophila* males by the MANN-WHITNEY-U-Test (Mann U) of their second run with a visual object and their second run without a visual object.

Group	U	Z	2*1 sided exact p
**CS 2nd run (24 h) + *vis.obj.* vs. CS 2nd run (24 h) +*no obj.***	111.50	3.9000	$<10^{-5}$
H24*>TNT 2nd run (24 h) + *vis.obj.* vs. *H24*>TNT 2nd run (24 h) +*no obj.	41.00	3.6966	0.0001
c305a*>TNT 2nd run (24 h) + *vis.obj.* vs. *c305a*>TNT 2nd run (24 h) + *no obj.	141.00	2.7132	0.0061
mb247*>TNT 2nd run (24 h) + *vis.obj.* vs. *mb247*>TNT 2nd run (24 h) + *no obj.	94.00	0.7673	0.4610
**c739>TNT 2nd run (24 h) + *vis.obj.* vs. c739>TNT 2nd run (24 h) + *no obj.***	171.50	-0.7709	0.4450

Supplementary Table 9 | Statistical analysis for Figure S2.

Tab. 9a refers to data presented in Figure S2a.

p-values of the KOLMOGOROV-SMIRNOV-Test (KS); LILLIFORS-Tests (Lillif) and SHAPIRO-WILK-Test (SW).

	N	Std.Dev.	p KS	p Lillif	p SW
**Pretest CS**	40	0.7910	< 0.01	< 0.01	$< 10^{-5}$
**Test CS Group Stress with sucrose**	20	0.9515	> 0.20	< 0.05	= 0.0459
**Test CS Group Stress no sucrose**	20	1.1910	> 0.20	< 0.05	= 0.0387

Comparison of the unpaired median data by the KRUSKAL-WALLIS-TEST (K-W) of climbing attempts.
Upper row: multiple p-values; lower row: z-values.

	(2)	(3)
K-Wallis-Test: H(2. N=80)=41.2017 $p< 10^{-5}$		
**Pretest (1)**<br>**CS**<br>R: 49.913	~1.0000<br>0.0452	$< 10^{-5}$<br>5.8710
**Pretest (2)**<br>**CS/ group stress with sucrose**<br>R: 49.625	--	$< 10^{-5}$<br>5.0453
**Pretest (3)**<br>**CS/ group stress no sucrose**<br>R: 12.550	--	--

Tab. 9b refers to data presented in Figure S2b.

p-values of the KOLMOGOROV-SMIRNOV-Test (KS); LILLIFORS-Tests (Lillif) and SHAPIRO-WILK-Test (SW).

	N	Std.Dev.	p KS	p Lillif	p SW
**Pretest Group 1 (CS)**	24	0.8165	< 0.10	< 0.01	= 0.0006
**Pretest Group 2 (CS)**	24	0.8297	< 0.01	< 0.01	= 0.0004
**Pretest Group 3 (CS)**	24	0.8502	< 0.15	< 0.01	= 0.0011
**Test 1 Group 1 (CS > stressed)**	23	1.1123	> 0.20	< 0.05	= 0.0040
**Test 1 Group 2 (CS > stressed)**	24	1.0135	> 0.20	< 0.05	= 0.0385
**Test 1 Group 3 (CS > stressed)**	24	1.1516	> 0.20	< 0.05	= 0.0479
**Test 2 (stressed) Group 1 + Sucrose**	23	1.1524	< 0.15	< 0.01	= 0.0163
**Test 2 (stressed) Group 2 + H_2O**	24	1.0632	< 0.15	< 0.01	= 0.0163
**Test 2 (stressed) Group 3 + Arabinose**	24	1.2730	> 0.20	< 0.05	= 0.1280

Comparison of the unpaired median data by the KRUSKAL-WALLIS-TEST (K-W).
Upper row: multiple p-values; lower row: z-values.

	(2)	(3)	(4)	(5)	(6)	(7)	(8)	(9)
K-Wallis-Test: $H(8\ N= 213)= 150.0822\ p< 10^{-5}$								
**Pretest (1)**<br>**Group 1 (CS)**<br>R: 154.00	~1.0000<br>0.5258	~1.0000<br>0.0796	$< 10^{-5}$<br>5.7285	$< 10^{-5}$<br>5.8349	$< 10^{-5}$<br>5.6335	~1.0000<br>0.2575	$< 10^{-5}$<br>6.0024	~1.0000<br>0.8063
**Pretest (2)**<br>**Group 2 (CS)**<br>R: 163.35	--	~1.0000<br>0.4461	$< 10^{-5}$<br>6.2487	$< 10^{-5}$<br>6.3607	$< 10^{-5}$<br>6.1593	~1.0000<br>0.7776	$< 10^{-5}$<br>6.5281	~1.0000<br>1.3264
**Pretest (3)**<br>**Group 3 (CS)**<br>R: 155.42	--	--	$< 10^{-5}$<br>5.8073	$< 10^{-5}$<br>5.9145	$< 10^{-5}$<br>5.7131	~1.0000<br>0.3363	$< 10^{-5}$<br>6.0820	~1.0000<br>0.8850
**Test 1/Stress (4)**<br>**Group 1 (CS)**<br>R: 50.978	--	--	--	~1.0000<br>0.0440	~1.0000<br>0.1553	$< 10^{-5}$<br>5.4138	~1.0000<br>0.2096	$< 10^{-5}$<br>4.8707
**Test 1/Stress (5)**<br>**Group 2 (CS)**<br>R: 50.188	--	--	--	--	~1.0000<br>0.2014	$< 10^{-5}$<br>5.5150	~1.0000<br>0.1674	$< 10^{-5}$<br>4.9662
**Test 1/Stress (6)**<br>**Group 3 (CS)**<br>R: 53.771	--	--	--	--	--	$< 10^{-5}$<br>5.3158	~1.0000<br>0.3689	0.0001<br>4.7670
**Test 2/Stress (7)**<br>**Group 1 + Sucrose**<br>R: 149.37	--	--	--	--	--	--	$< 10^{-5}$<br>5.6807	~1.0000<br>0.5431
**Test 2/Stress (8)**<br>**Group 2 + H_2O**<br>R: 47.208	--	--	--	--	--	--		$< 10^{-5}$<br>5.1319
**Test 2/Stress (9)**<br>**Group 2 + Arabinose**<br>R: 139.50	--	--	--	--	--	--	--	

Tab. 9c refers to data presented in Figure S2c.

p-values of the KOLMOGOROV-SMIRNOV-Test (KS); LILLIFORS-Tests (Lillif) and SHAPIRO-WILK-Test (SW).

	N	Std.Dev.	p KS	p Lillif	p SW
**Group Sucrose**	8	0.4711	> 0.20	< 0.15	= 0.0245
**Group H_2O**	8	0.4244	> 0.20	> 0.20	= 0.9998
**Group Sucrose + αMTP**	11	0.2603	> 0.20	> 0.20	= 0.5193

Comparison of the normally distributed data of the 5HT-quantification in the ellipsoid body by the unpaired T-TEST of *Drosophila* males of the stressed and control group.

Group	t	F	p
**Group Sucrose**<br>**vs.**<br>**Group H_2O**	-2.2659	1.2318	0.0398
**Group Sucrose**<br>**vs.**<br>**Group Sucrose + αMTP**	2.6996	3.2753	0.0152
**Group H_2O**<br>**vs.**<br>**Group Sucrose + αMTP**	-0.3413	2.6589	0.7371

Tab. 9d refers to data presented in Figure S2d.

p-values of the KOLMOGOROV-SMIRNOV-Test (KS); LILLIFORS-Tests (Lillif) and SHAPIRO-WILK-Test (SW).

	N	Std.Dev.	p KS	p Lillif	p SW
**H_2O**	8	0.4815	> 0.20	> 0.20	= 0.4393
**Sucrose**	8	1.4230	> 0.20	< 0.05	= 0.0230
**Sucrose + αMTP**	11	0.3387	> 0.20	< 0.15	= 0.2052

Comparison of the unpaired median data by the KRUSKAL-WALLIS-TEST (K-W).
Upper row: multiple p-values; lower row: z-values.

Genotype	(2)	(3)
K-Wallis-Test: H(2 N=27)= 10.5251 p< 0.0052		
**H_2O (1)**<br>R: 13.125	0.1219<br>2.0473	0.9234<br>0.0199
**Sucrose (2)**<br>R: 21.250	--	0.0038<br>3.2229
**Sucrose + αMTP (3)**<br>R: 9.364	--	--

Tab. 9e refers to data presented in Figure S2e.

p-values of the KOLMOGOROV-SMIRNOV-Test (KS); LILLIFORS-Tests (Lillif) and SHAPIRO-WILK-Test (SW).

	N	Std.Dev.	p KS	p Lillif	p SW
**GFP intensity in α-lobes Control**	12	996194.8	> 0.20	> 0.20	= 0.3615
**GFP intensity in α lobes Stressed group**	12	750375.7	> 0.20	< 0.10	= 0.1278
**GFP Intensity in β-lobes Control**	12	957297.6	> 0.20	> 0.20	= 0.5101
**GFP Intensity in β-lobes Stressed group**	13	702174.6	> 0.20	< 0.20	= 0.5537
**GFP Intensity in the EB Control**	12	896035.9	> 0.20	> 0.20	= 0.4320
**GFP Intensity in the EB Stressed group**	13	960263.4	> 0.20	> 0.20	= 0.2081

Comparison of the normally distributed data by the unpaired T-TEST (K-W) of the GFP Intensity in different brain compartments of the stressed flies and the control group.

Group	T	F	p
**Stress vs. Control (in α-lobes)**	-0.4479	1.7625	0.6586
**Stress vs. Control (in β-lobes)**	-1.6970	1.8587	0.1032
**Stress vs. Control (in the EB)**	-1.9030	1.1485	0.0696

Supplementary Table 10 | Statistical analysis for Figure S4.

Tab. 10a refers to data presented in Figure S4a.

p-values of the KOLMOGOROV-SMIRNOV-Test (KS); LILLIFORS-Tests (Lillif) and SHAPIRO-WILK-Test (SW).

	**N**	**Std.Dev.**	**p KS**	**p Lillif**	**p SW**
**Pretest**<br>***mb247*>>GAL80^{ts}>5HT1A^{RNAi}**	21	1.2209	> 0.20	< 0.01	= 0.0683
**Pretest**<br>***mb247*>>GAL80^{ts}>5HT1A^{RNAi}**	21	0.9284	< 0.20	< 0.01	= 0.0214
**Test 1**<br>***mb247*>>GAL80^{ts}>5HT1A^{RNAi}**<br>**Stressed Group (18°C)**	21	1.0556	< 0.15	< 0.01	= 0.0594
**Test 1**<br>***mb247*>>GAL80^{ts}>5HT1A^{RNAi}**<br>**Stressed Group (29°C)**	21	1.1609	< 0.10	< 0.01	= 0.0216
**Test 2 (sucrose)**<br>***mb247*>>GAL80^{ts}>5HT1A^{RNAi}**<br>**Stressed Group (18°)**	20	1.2763	> 0.20	< 0.15	= 0.3753
**Test 2 (sucrose)**<br>**mb247>>GAL80^{ts}>5HT1A^{RNAi}**<br>**Stressed Group (29°C)**	21	1.1106	> 0.20	< 0.05	= 0.1070

Comparison of the unpaired median data by the KRUSKAL-WALLIS-TEST (K-W).
Upper row: multiple p-values; lower row: z-values.

**Genotyp**	**(2)**	**(3)**	**(4)**	**(5)**	**(6)**
K-Wallis-Test: H(5 N=125)= 85.9224 p< 10^{-5}					
**Pretest/Stress (1)**<br>***mb247*>>GAL80^{ts}>5HT1A^{RNAi}**<br>R: 95.143	~1.0000<br>0.3237	< 10^{-5}<br>5.1366	< 10^{-5}<br>5.6562	~1.0000<br>0.2997	< 10^{-5}<br>5.7073
**Pretest/Stress (2)**<br>**mb247>>GAL80^{ts}>5HT1A^{RNAi}**<br>R: 91.524	--	< 10^{-5}<br>4.8129	< 10^{-5}<br>5.3325	~1.0000<br>0.0110	< 10^{-5}<br>5.3836
**Test 1/Stress (18°C) (3)**<br>***mb247*>>GAL80^{ts}>5HT1A^{RNAi}**<br>R: 37.714	--	--	~1.0000<br>0.5196	< 10^{-5}<br>4.7738	~1.0000<br>0.5707
**Test 1/Stress (29°) (4)**<br>***mb247*>>GAL80^{ts}>5HT1A^{RNAi}**<br>R: 31.905	--	--	--	< 10^{-5}<br>5.2870	~1.0000<br>0.0511
**Test 2/Stress (18°C)(5)**<br>***mb247*>>GAL80^{ts}>5HT1A^{RNAi}**<br>R: 91.750	--	--	--	--	< 10^{-5}<br>5.3375
**Test 2/Stress (29°C) (6)**<br>***mb247*>>GAL80^{ts}>5HT1A^{RNAi}**<br>R: 31.333	--	--	--	--	--

Tab. 10b refers to data presented in Figure S4b

p-values of the KOLMOGOROV-SMIRNOV-Test (KS); LILLIFORS-Tests (Lillif) and SHAPIRO-WILK-Test (SW).

	N	Std.Dev.	p KS	p Lillif	p SW
**Pretest** ***mb247*>>GAL80^{ts}>5HT1B^{RNAi}**	18	0.7048	< 0.10	< 0.01	= 0.0056
**Pretest** ***mb247*>>GAL80^{ts}>5HT1B^{RNAi}**	18	0.8498	< 0.10	< 0.01	= 0.0159
**Test 1** ***mb247*>>GAL80^{ts}>5HT1B^{RNAi}** **Stressed Group (18°C)**	18	1.1827	< 0.15	< 0.01	= 0.0865
**Test 1** ***mb247*>>GAL80^{ts}>5HT1B^{RNAi}** **Stressed Group (29°C)**	18	0.9785	> 0.20	< 0.05	= 0.0267
**Test 2 (sucrose)** ***mb247*>>GAL80^{ts}>5HT1B^{RNAi}** **Stressed Group (18°)**	17	0.8703	< 0.15	< 0.01	= 0.0139
**Test 2 (sucrose)** ***mb247*>>GAL80^{ts}>5HT1B^{RNAi}** **Stressed Group (29°C)**	17	1.1726	> 0.20	< 0.05	= 0.0997

Comparison of the unpaired median data by the KRUSKAL-WALLIS-TEST (K-W).
Upper row: multiple p-values; lower row: z-values.

Genotyp	(2)	(3)	(4)	(5)	(6)
K-Wallis-Test: H(5 N=106)= 39.0988 p< 10^{-5}					
**Pretest/Stress (1)** ***mb247*>>GAL80^{ts}>5HT1B^{RNAi}** R: 60.972	~1.0000<br>0.5584	0.0001<br>4.5131	~1.0000<br>0.0379	~1.0000<br>0.0313	~1.0000<br>0.8032
**Pretest/Stress (2)** ***mb247*>>GAL80^{ts}>5HT1B^{RNAi}** R: 55.250	--	0.0011<br>3.9548	~1.0000<br>0.5963	~1.0000<br>0.5191	~1.0000<br>1.3536
**Test 1/Stress (18°C) (3)** ***mb247*>>GAL80^{ts}>5HT1B^{RNAi}** R: 14.722	--	--	0.0001<br>4.5511	0.0002<br>4.4169	< 10^{-5}<br>5.2514
**Test 1/Stress (29°) (4)** ***mb247*>>GAL80^{ts}>5HT1B^{RNAi}** R: 61.361	--	--	--	~1.0000<br>0.0687	~1.0000<br>0.7658
**Test 2/Stress (18°C)(5)** ***mb247*>>GAL80^{ts}>5HT1B^{RNAi}** R: 60.647	--	--	--	--	~1.0000<br>0.8228
**Test 2/Stress (29°C) (6)** ***mb247*>>GAL80^{ts}>5HT1B^{RNAi}** R: 69.324	--	--	--	--	--

Supplementary References

S1. Sweeney, S. T., Broadie, K., Keane, J., Niemann, H. & O'Kane, C. J. Targeted expression of tetanus toxin light chain in *Drosophila* specifically eliminates synaptic transmission and causes behavioral defects. *Neuron* **14,** 341-351 (1995).

S2. Aso, Y. *et al*. The mushroom body of adult *Drosophila* characterized by GAL4 drivers. *J. Neurogenet*. **23,** 156-172 (2009).

S3. Gasque, G., Conway, S., Huang, J., Rao, Y. & Vosshall, L. B. Small molecule drug screening in *Drosophila* identifies the 5HT2A receptor as a feeding modulation target. *Sci. Rep*. **3,** srep02120 doi: 10.1038/srep02120 (2013).

S4. Yuan, Q., Lin, F., Zheng, X. & Sehgal, A. Serotonin modulates circadian entrainment in *Drosophila*. *Neuron* **47,** 115-127 (2005).

S5. McGuire, S. E., Le, P. T., Osborn, A. J., Matsumoto, K. & Davis, R. L. Spatiotemporal rescue of memory dysfunction in *Drosophila*. *Science* **302,** 1765-1768 (2003).
